# A *Gypsy* element contributes to the nuclear retention and transcriptional regulation of the resident lncRNA in locusts

**DOI:** 10.1080/15476286.2021.2024032

**Published:** 2022-01-22

**Authors:** Xia Zhang, Ya′Nan Zhu, Bing Chen, Le Kang

**Affiliations:** a State Key Laboratory of Integrated Management of Pest Insects and Rodents, Institute of Zoology, Chinese Academy of Sciences, Beijing, China; b CAS Center for Excellence in Biotic Interactions, University of Chinese Academy of Sciences, Beijing, China; c School of Life Sciences, Hebei University, Baoding, China; d Beijing Institute of Life Sciences, Chinese Academy of Sciences, Beijing, China

**Keywords:** lncRNA, transposable element, SRSF2, migratory locust, *Ty3*/*Gypsy* element, nuclear retention

## Abstract

The majority of long noncoding RNAs (lncRNAs) contain transposable elements (TEs). *PAHAL*, a nuclear-retained lncRNA that is inserted by a *Gypsy* retrotransposon, has been shown to be a vital regulator of phenylalanine hydroxylase (*PAH*) gene expression that controls dopamine biosynthesis and behavioural aggregation in the migratory locust. However, the role of the *Gypsy* retrotransposon in the transcriptional regulation of *PAHAL* remains unknown. Here, we identified a *Gypsy* retrotransposon (named *Gypsy* element) as an inverted long terminal repeat located in the 3′ end of *PAHAL*, representing a feature shared by many other lncRNAs in the locust genome. The embedded *Gypsy* element contains a RNA nuclear localization signal motif, which promotes the stable accumulation of *PAHAL* in the nucleus. The *Gypsy* element also provides high-affinity SRSF2 binding sites for *PAHAL* that induce the recruitment of SRSF2, resulting in the *PAHAL*-mediated transcriptional activation of *PAH*. Thus, our data demonstrate that TEs provide discrete functional domains for lncRNA organization and highlight the contribution of TEs to the regulatory significance of lncRNAs.

## Introduction

Thousands of long noncoding RNAs (lncRNAs) have been extensively described in many species and act as vital and flexible cellular modulators to affect various fundamental biological processes via diverse mechanisms [1–4]. Functional diversification of lncRNAs is the foundation of the RNA-based regulatory mechanisms that highlight the closer relationship between the degree of organic and behavioural complexity and the number of lncRNA species, rather than the number of protein-coding genes [5]. Recent studies have reported that several lncRNAs participate in neuronal development and cognitive and behavioural regulation [6–10]. During phase changes from the gregarious (G) to the solitarious (S) states in the migratory locust, the dopamine (DA) pathway in coding genes and noncoding RNAs plays a crucial role in the regulation of locust aggregative behaviour. The phenylalanine hydroxylase gene (*PAH*, also referred to as *henna* in *Drosophila* and *Locusta*) is a key gene for DA synthesis in this pathway [11]. *PAH* transcriptional regulation is essential for the locust behavioural changes [11]. The posttranscriptional modification of this gene by miRNA 133 is involved in locust behavioural phase changes [12]. Recent findings demonstrate that *PAHAL*, a *PAH* lncRNA arranged in the sense orientation, is unique as a modulator of reversible locust behavioural changes; *PAHAL* functions by accelerating ancestral *PAH* gene expression, resulting in DA production in the locust brain [13].

Mechanistically, *PAHAL* acts as a nuclear lncRNA to recruit serine/arginine-rich splicing factor 2 (SRSF2) to the *PAH* proximal promoter, promoting *PAH* transcriptional activation [13]. Further analysis showed that the RNA nuclear localization signal motif (NLS) and the SRSF2 binding site are located at the 3′ terminus of *PAHAL*. The 3′ end sequence of *PAHAL* plays a vital role in the transcriptional regulation function and contains a long terminal repeat (LTR) of the *Ty3*/*Gypsy* retroelement [13]. Therefore, *PAHAL* is a transposable element (TE)-embedded lncRNA.

Similar to proteins, the primary sequence of a lncRNA comprises ‘domains’ or discrete elements that modulate specific aspects of lncRNA activity, such as molecular interactions and subcellular localization [1,14–16]. TEs could be a possible source of lncRNA domains, providing a structured RNA platform and sequence features for the biogenesis and subcellular localization of the resident lncRNA, as well as the modulation of lncRNA downstream gene expression [14,16–20]. In particular, *Ty3*/*Gypsy* retroelements constitute a large family of LTR retrotransposons and are widely distributed in plants, fungi, and animals [21]. Insertions of LTR remnants in and around the 5′ UTRs, introns and 3′ UTRs of genes may alter their structure, function, stabilization and evolution by providing new regulatory units, such as transcription factor binding sites, alternative polyadenylation sites, and alternative splicing sites [22,23]. A large fraction of LTRs lost their transposability during evolution through deletion of their internal coding genes [24]. These LTRs, as embedded sequences, are abundant in mature lncRNAs and account for a significant portion of the total lncRNA sequence [18]. Some studies have identified that LTRs are highly enriched at transcription start sites of long intergenic noncoding RNAs (lincRNAs) to promote transcription by providing transcription factor (TF) binding sites [20]. For example, *LTR7/HERVH* elements seed NANOG, OCT4, and SOX2 binding sites to enhance *lncRNA-RoR* and *lncRNA-ES3* transcription in human embryonic stem cells (ESCs) [18]. The *invSINEB2* element, as an effector domain, is embedded in the lncRNA *AS Uchl1*, which is known to be involved in the degeneration of dopaminergic neurons [25–27]. The neurodegenerative disease-associated lncRNA *Malat1* recruits hnRNPK to maintain nuclear speckles through the embedded *SINEB1* [7]. Moreover, the exonization of LTRs may be one of the possible ways of regulating phenotypic plasticity of the locusts [28,29]. While the contribution of TEs to lncRNA regulation is evident, the specific mechanism underlying TE-mediated regulation largely remains elusive.

Here, we showed that the residence of a noncanonical *Gypsy* element in a lncRNA is a common feature of many other lncRNAs in the locust genome. We found that the *Gypsy* element provides an NLS and three tandem SRSF2 high-affinity sites (exonic-splicing enhancer, i.e. ESE) for *PAHAL*. The *Gypsy* element determines nuclear retention, prolongs the half-life of *PAHAL*, and affects the affinity between *PAHAL* and SRSF2 via the NLS and ESEs, resulting in transcriptional activation of *PAH*. These results demonstrate that the embedded *Gypsy* element is a functionally important motif for *PAHAL* regulation. Our findings also provide a mechanistic explanation for the elaborate transcriptional regulation, in which a TE confers the resident lncRNA with the ability to serve as a protein binding motif to modulate protein activity, for transcriptional activation.

## Materials and methods

### Animals

The locusts were maintained strictly under standard conditions established by previous reports [13,30]. Briefly, approximately five hundred G locusts were reared in a large cage (40 cm × 40 cm × 40 cm). The S locusts were cultured individually in white metal boxes (10 cm × 10 cm × 25 cm) supplied with charcoal-filtered fresh air. The locust colonies were reared under a 14:10 light/dark photoperiod at 30 ± 2°C and fed fresh wheat seedlings and bran.

### Cells

*Drosophila* S2 cells (Gibco, NY, USA; R69007) were grown in SFX-insect medium (HyClone, Logan, UT; SH30278.02) at 28°C. The SRSF2 protein-depleted mouse embryonic fibroblast line (SRSF2-MEFs) is an endogenous SRSF2-KO system of mouse cells that can turn off endogenous SRSF2 transcription upon addition of doxycycline (DOX) to the culture medium. We used SRSF2-MEFs to study the role of the embedded *Gypsy* element in the interaction of SRSF2 with *PAHAL*. SRSF2-MEFs were maintained in DMEM (Gibco, NY, USA; C11965500BT) supplemented with tet-free FBS (Clontech, CA, USA; 631,106) in standard culture incubators (37°C, humidified 5% CO_2_/95% air). SRSF2-MEFs were treated with 10 µg/mL DOX (Sigma, MO, USA; D9891-1 G) for 1 day to deplete endogenic SRSF2 transcription (DOX+) [31].

### RNA isolation and qPCR

Freshly harvested tissues were stored in liquid nitrogen before RNA preparation. Cultured cells were collected and then washed twice in PBS before RNA extraction. Total RNA was extracted according to the manufacturer’s instructions for TRIzol reagent (Invitrogen, CA, USA, 15,596,018). cDNA was synthesized with a Fastking RT Kit (With gDNase) (Tiangen, Beijing, China; KR116). qPCR was performed with Talent qPCR PreMix (SYBR Green) (Tiangen, Beijing, China; FP209) in a LightCycler 480 instrument (Roche, Mannheim, Germany). All the PCR products were verified through sequencing before qPCR. The housekeeping gene ribosomal protein 49 (RP49) was used as an internal control for gene expression normalization [13,30,32]. Five to eight biological replicates were prepared for each treatment. All primers are listed in Supplementary Table S1.

### Isolation and crowding of locusts

Standard procedures of isolation and crowding of locusts were performed as previously described with some modifications [11,13,33]. Briefly, the locusts were separately reared from G nymphs in solitary rearing cages under standard conditions. The locusts were crowded by introducing 10 labelled S nymphs into an optic Perspex box (10 cm × 10 cm × 10 cm) that contained 20 G nymphs. After 0, 4, 8, 16 or 32 h of treatment, the locust brains were dissected and immediately put into liquid nitrogen for RNA preparation. Equal numbers of male and female insects were sampled for each biological replicate at the same developmental stage.

### Northern blot analysis

Northern blot analysis was performed as previously described with slight amendment [34]. A portion (25 μg) of DNase I-treated total RNA was extracted using TRIzol reagent. Denaturing formaldehyde agarose gels (1%) were used for sample RNA separation by electrophoresis. The separated RNA was transferred onto a BrightStar Plus membrane (Ambion, Vilnius, Lithuania, AM10102) by capillary action using Alkaline transfer buffer [5× SSC (Invitrogen, N.Y., USA, AM9763), 10 mM NaOH] overnight at room temperature (RT) and was immediately UV cross-linked for 300 s at 120 mJ/cm^2^ to reduce RNA degradation. The membrane was pre-hybridized for 1 h at 37°C in ULTRAhyb-Oligo Hybridization buffer (Invitrogen, Vilniue, Lithuania, AM8663). *PAHAL-PAH* RNA probe, which covered the overlapping sequence of *PAHAL* and *PAH*, was synthesized and labelled with biotin using T7 RNA Polymerase kit (Promega, WI, USA; P2075). The 3′ biotin-labelled *U6* DNA probe [35] was synthesized as endogenous control by Thermo Fisher (BJ, China). Then, the membrane was hybridized with *PAHAL-PAH* RNA probe and *U6* DNA probe at 37°C overnight. After two washes using the washing buffer (2× SSC, 0.5% SDS) at 37°C, the blots were detected by Chemiluminescent Nucleic Acid Detection Module (Pierce, CA, USA; 89,880).

### Reporter and expression plasmid construction

Different constructs were prepared: The full-length *PAHAL¯* sequence (labelled as *PAHAL¯*), *PAHAL* with the *Gypsy* retroelement deleted (i.e.*PAHAL*^Δ*Gypsy*^), *PAHAL* with the NLS deleted (*PAHAL*^ΔNLS^), the *PAHAL¯* sequence with an artificial insertion of the *Gypsy* element immediately preceding the poly(A) sequence of *PAHAL¯* (i.e. *PAHAL¯^Gypsy+^*), and *PAHAL* with the mutation of the tandem ESEs (i.e. MT-ESEs-*PAHAL*); they were cloned into the pcDNA3.1 (+) vector (overexpression vector for SRSF2-MEFs; Invitrogen, CA, USA; V79020) and pAc5.10/V5-His A vector (overexpression vector for *Drosophila* S2 cells; Invitrogen, CA, USA; V4110-20). pGL4.10-P + 5′UTR (with the *PAH* promoter fused to a firefly luciferase reporter), pcDNA3.1/*PAHAL* (*PAHAL* overexpression vector for SRSF2-MEFs), pAc5.10/V5-His A/*PAHAL* (*PAHAL* overexpression vector for *Drosophila* S2 cells), pcDNA3.1/*lacz* (negative control vector for SRSF2-MEFs), pAc5.10/V5-His A/*lacz* (negative control vector for *Drosophila* S2 cells), pcDNA3.1/V5-His/*SRSF2* ORF (SRSF2 overexpression vector for SRSF2-MEFs) and pAc5.10/V5-His A/*SRSF2* ORF (SRSF2 overexpression vector for *Drosophila* S2 cells) were constructed and described previously [13]. These vectors were transfected into *Drosophila* S2 cells and SRSF2-MEFs.

### RNA decay rate assay

For *in vitro* experiments, the *PAHAL, PAHAL*^Δ*Gypsy*^, *PAHAL¯^Gypsy+^* or *PAHAL*^ΔNLS^ vector was transfected into SRSF2-MEFs using Lipofectamine 3000 (Invitrogen, CA, USA; L3000015). The second day after transfection, transcription was halted for 1 to 7 h or 1 to 4 h by adding 5 mg/mL actinomycin D (Act D) (Sigma, MO, USA; A4262-5 mg) to obtain a final concentration of 1 μg/mL. For *in vivo* experiments, Act D was dissolved at a concentration of 1 mg/mL in DMSO and then diluted to 0.4 μg/μL in PBS. The brains of G locusts were microinjected with 69 nL of this Act D solution for 1 to 4 h. Cells and locust brains were harvested in TRIzol at different time points to assess the decay rate of *PAHAL, PAHAL¯, PAHAL*^Δ*Gypsy*^, *PAHAL*^ΔNLS^, *PAHAL¯^Gypsy+^* or MT-ESEs-*PAHAL* RNA. Half-lives were calculated using one-phase exponential decay [36].

### Cell fractionation experiment

Nuclear fractionation experiments form brains or cells were performed as previously reported [13,37]. Twenty nymphal brains or 2 × 10 ^7^cells were harvested by centrifugation and homogenized in cold lysis buffer [1× PBS supplemented with 0.2% IGEPAL CA-630 (Sigma, MO, USA; I8896-50 ml), 1× proteinase inhibitor (Pierce MA, USA; 88,266) and RNase inhibitor (Promega, WI, USA; N2111S)]. The cell residue in the homogenate was removed by centrifugation at 30 × *g* for 2 min at 4°C. The nuclear pellet was obtained by centrifugation at 425 × *g* for 15 min at 4°C. The residual nuclei were removed by centrifugation at 2000 × *g* for 10 min at 4°C to obtain the cytoplasmic fraction in the supernatant. The cell fractionation was stored at −80°C prior to RNA extraction and the RNA immunoprecipitation (RIP) assay.

### RNA fluorescence in situ hybridization (FISH)

To determine whether the embedded *Gypsy* element affects the nuclear retention of *PAHAL*, fluorescence in situ hybridization (FISH) experiments were performed as previously described with some modifications [13,37]. Universal biotinylated RNA probes were designed for *PAHAL, PAHAL*^Δ*Gypsy*^ and *PAHAL*^ΔNLS^ and then synthesized by using a T7 RNA Polymerase Kit (Promega, WI, USA, P2075). SRSF2-MEFs were seeded onto 6-well plates (Corning, NY, USA) and then transfected with pcDNA3.1/*PAHAL*, pcDNA3.1/*PAHAL*^Δ*Gypsy*^ and pcDNA3.1/*PAHAL*^ΔNLS^. The cells were harvested and fixed in 4% (wt/vol) paraformaldehyde for 1 h at RT. The fixed cells were permeabilized with PBST (0.5% Triton X-100 in 1× PBS) for 10 min at RT and then digested with 20 µg/mL proteinase K (Invitrogen, CA, USA; AM2548) at 37°C for 15 min. The cell pelleted was incubated with prehybridization buffer (Wuhan Boster, Wuhan, China; AR0152) at 37°C for 30 min. The cells were hybridized with probes (5 ng/µL) at 37°C overnight and then blocked with blocking buffer (2% BSA in 0.2× SSC) at 4°C for 20 min. Next, the cells were incubated with streptavidin–HRP (1:100) for 1 h at RT and then washed three times with PBS. The fluorescent biotin signal was detected with a TSA Fluorescein System (Perkin-Elmer, MA, USA; NEL701A001KT). The cells were centrifuged, resuspend in Antifade Mounting Medium (Wuhan Boster, Wuhan, China; AR1109), and then dropped onto slides. Images were captured with an LSM 710 confocal fluorescence microscope (Zeiss, Oberkochen, Germany) at 63× magnification. Supplementary Table S1 lists the primers used for FISH probe synthesis.

### RIP assay

pcDNA3.1/V5-His/*SRSF2* ORF was cotransfected with pcDNA3.1/*PAHAL*, pcDNA3.1/*PAHAL*^Δ*Gypsy*^ or pcDNA3.1/ *PAHAL*^ΔNLS^ into SRSF2-MEFs that had been depleted of mouse endogenous SRSF2 by adding DOX for one day to test whether the embedded *Gypsy* element affects the binding of SRSF2 with *PAHAL in vitro*. After 3 days, 2 × 10^7^ SRSF2-MEFs were harvested using a cell scraper. Nuclei were isolated for the RIP experiment *in vitro*. The binding affinity of SRSF2 to *PAHAL¯* RNA *in vivo* was tested by performing the RIP assay on brain tissues. Nuclei were isolated from fifty brains for the RIP experiment *in vivo*.

A Magna RIP Quad RNA-Binding Protein Immunoprecipitation Kit (Millipore, CA, USA; 17–704) was used to perform the RIP assay. The nuclear pellet was lysed in ice-cold RIP lysis buffer spiked with 1× proteinase inhibitor and RNase inhibitor and stored at −80°C overnight. Magnetic beads were sensitized by preincubation with 5 µg of V5 antibody (Invitrogen, CA, USA; R96025) or normal mouse IgG (Millipore, CA, USA; CS200621) for 30 min at RT with rotation to form the bead–antibody complex. The supernatant of the lysate from the centrifugation was added to the bead–antibody complex. The mixture was coincubated overnight at 4°C with rotation to bind the candidate RNAs. Thereafter, 10 µL of the supernatant was sampled as the input. Candidate RNAs in the immunoprecipitate and input were analysed through qPCR.

### RNA pulldown and Western blot analysis

RNA pulldown experiments were conducted according to the manufacturer’s recommendations for the Magnetic RNA–Protein Pull-Down Kit (Thermo Fisher Scientific, CA, USA; 20,164) with some modifications. Briefly, the DNA templates of the RNA probes for a series of ESEs with mutations in the embedded *Gypsy* element of *PAHAL* were synthesized by PolePolar Biotechnology Co., Ltd., Beijing, China. Biotinylated RNA probes were transcribed with a T7 RNA Polymerase Kit (Promega, WI, USA; P2075). In addition, endogenous SRSF2 was turned off in SRSF2-MEFs by treatment with DOX for 1 day. Subsequently, the cells were transfected with the pcDNA3.1/V5-His/*SRSF2* ORF vector.

On the third day after transfection, the cells were lysed to extract total protein by using lysis buffer [T-PER Tissue Protein Extraction Reagent (Pierce, CA, USA; 78,510) containing 1× Halt Protease Inhibitor Cocktail, EDTA-free (Pierce, CA, USA; 87,785) and 1× RNase inhibitor (Promega, WI, USA; N2111S)]. The total protein was incubated with biotinylated RNA probes for 1 h at 4°C with rotation. RNA-binding proteins were analysed by Western blotting.

The proteins were isolated by a 10% NuPAGER Bis-Tris gel (Invitrogen, CA, USA; NP0315BOX) and subsequently transferred to PVDF membranes. The membranes were blocked with 5% (wt/vol) skim milk for 1 h at RT. SRSF2 was stained using a V5 tag monoclonal antibody (Invitrogen, CA, USA; R96025; 1:5,000) and a secondary antibody (Easybio, Beijing, China; BE0102-100; 1:5,000) and was detected with a SuperSignal West Femto Substrate Trial Kit (Pierce, CA, USA; 34,094).

### Luciferase assay

To test whether the embedded *Gypsy* element affected the transcriptional activation function of *PAHAL*, we performed luciferase assays. Lipofectamine 3000 was used for plasmid delivery into cells that were expanded on 48-well plates (Corning, NY, US) for one night. The reporter plasmid (pGL4.10-P + 5′UTR, 10 ng) was cotransfected with 200 ng of the expression plasmid or negative control vector into cells with 5 ng of the internal control vector pRL-TK (Promega, WI, USA; E2241) to express Renilla luciferase. Both firefly and Renilla luciferase activities were measured using a dual-luciferase reporter assay system (Promega, WI, USA; E1960) at 30 h after incubation.

### Bioinformatics and statistical analysis

The sequence motif of the NLS was WNNNNSNNAGCCC (W = A/T, S = G/C) [38]. The sequence of the SRSF2 high-affinity ESE site was WSSNGYY (W = A/T, S = G/C Y = C/T) [39,40]. Data from the tissue expression experiment, mutational analysis of SRSF2 affinity and the nuclear retention analysis of *PAHAL* were analysed through ANOVA and then by post hoc Tukey’s *b*-test for multiple comparisons. Differences in gene expression and other values between treatments were analysed by using independent-sample Student’s *t* tests. The data are described as the mean ± SEM unless stated otherwise. SPSS 21.0 (SPSS Inc., IL, USA) was used for all statistical analyses. The locust genome data are available at the following website: http://www.locustmine.org. The sequence for *PAHAL¯* has been deposited in GenBank under accession number KX962172. Numerical data that underlies graphs and sample image data have been uploaded to https://dataverse.harvard.edu/dataset.xhtml?persistentId=doi:10.7910/DVN/UETCO0.

## Results

### *Gypsy* element-embedded lncRNAs are widespread in the locust genome

**Figure 1.**
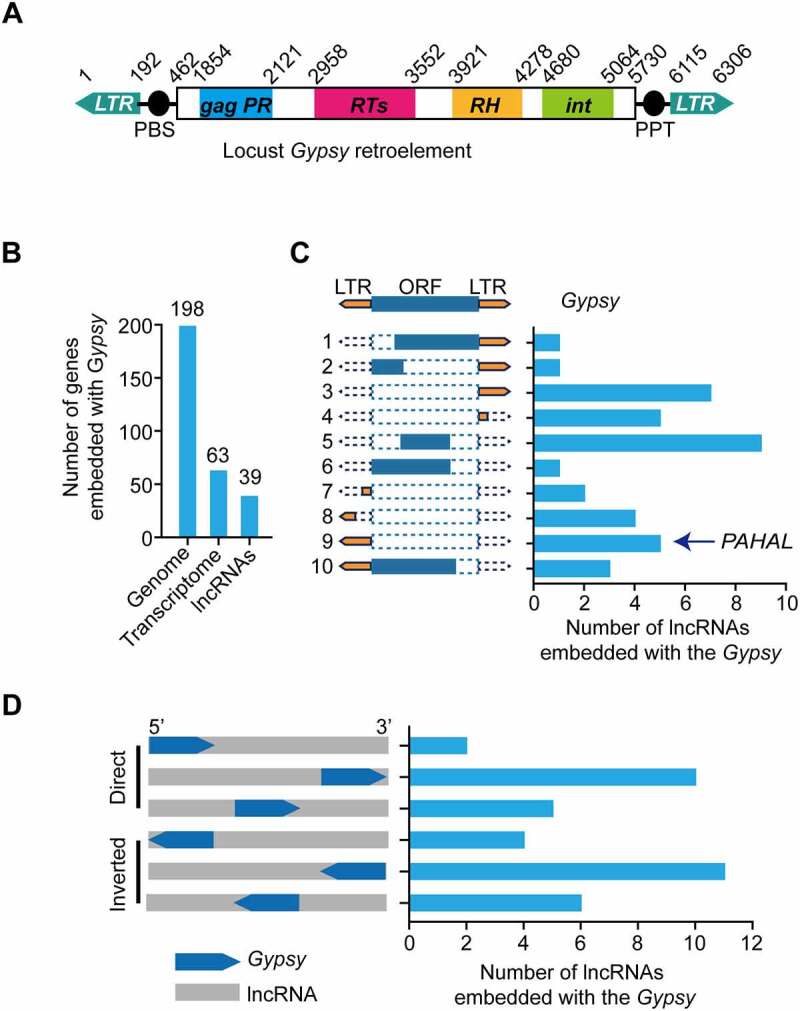
Contribution of embedded *Gypsy* elements to the diversification of lncRNAs in the locust genome. (a) Structure of a canonical *Gypsy* element (*Gypsy*-25 subfamily) in the locust genome. (b) Amounts of the embedded *Gypsy* elements transcribed with lncRNAs in the genome. (c) Structure and amounts of different embedded *Gypsy* elements present in locust lncRNAs. The structure of a *Gypsy* element in a lncRNA is illustrated in the left panel. The histogram in the right panel shows the number of each type of element present in lncRNAs. (d) Insertion profiles of *Gypsy* elements in locust lncRNAs. The location of the *Gypsy* element insertion in lncRNAs is illustrated in the left panel; 5′ and 3′ represent the lncRNA direction.

We previously defined a 2.6-kb lncRNA, *PAHAL*, which is involved in the feedback regulation of locust behavioural aggregation. *PAHAL* possesses a 217-nt LTR of the noncanonical *Ty3*/*Gypsy* retroelement (named *Gypsy* element) immediately preceding the poly(A) sequence [13]. Eighty-three subfamilies of locust *Ty3*/*Gypsy* retroelements are annotated in Repbase [41]. The *Gypsy* element contained in *PAHAL* belongs to the *Gypsy*-25 subfamily, the classic structure of which harbours an inverted pair of LTRs flanking the retrotransposon (Figure 1(a)).

In this study, we first investigated whether *Gypsy* element-embedded lncRNAs are ubiquitous in the locust genome. We scanned the locust genome and transcriptomes for the *Gypsy*-25 retroelements and the element-embedded lncRNAs. The locust genome contained 198 copies of the elements. Among them, 62 transcripts contained at least a partial element. A total of 38 *Gypsy*-embedded transcripts were identified in silico as lncRNAs (Figure 1(b)).

We annotated the structure of the elements embedded in the lncRNAs and found 10 different types of elements (Figure 1(c)). All the *Gypsy* element types that we identified were noncanonical. These elements were embedded in direct or inverted directions relative to the resident lncRNA and in different lncRNA regions, such as the 5′UTR, middle region, and 3′UTR (Figure 1(d)). In addition to *PAHAL*, at least four *PAHAL*-like lncRNAs were identified in the specified transcriptomes. Therefore, *Gypsy* element-embedded lncRNAs are common in the locust genome.

### The presence of the embedded *Gypsy* element is associated with *PAHAL* and *PAH* expression

**Figure 2.**
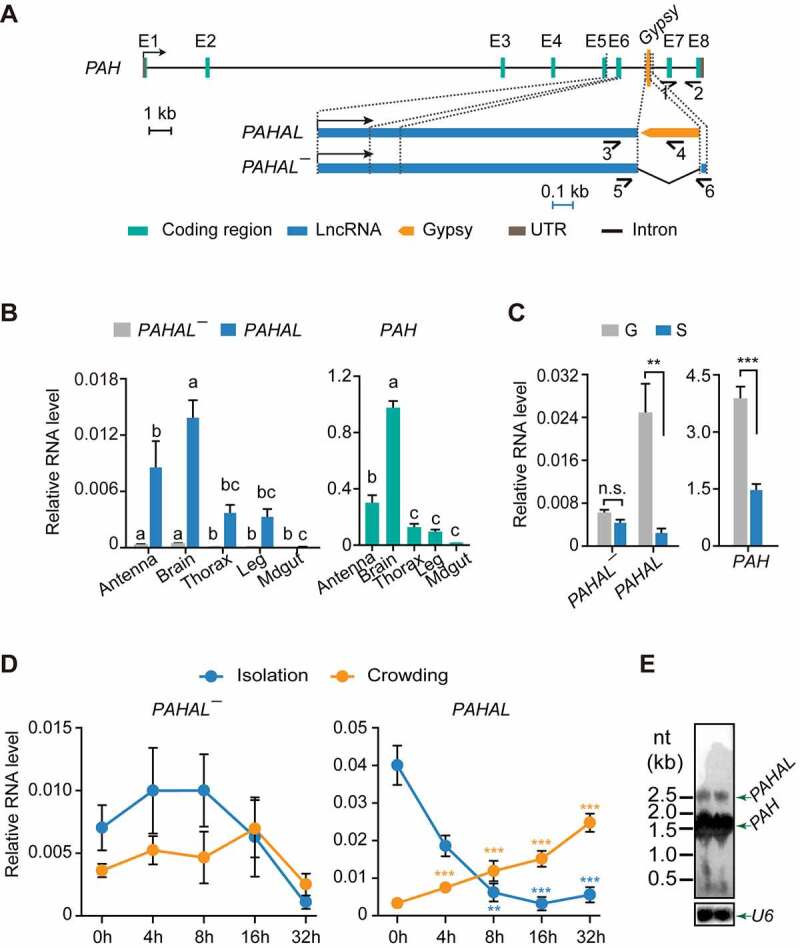
Presence of the *Gypsy* element in the lncRNA *PAHAL* and associated gene expression changes. (a) Gene and transcript structures of locust *PAH, PAHAL*, and *PAHAL¯*. The transcriptional orientations of *PAH, PAHAL* and *PAHAL¯* are labelled with bent arrows. The eight exons of the *PAH* transcript are indicated using ‘E 1–8’. The half-arrows indicate the strand-specific quantitative real-time (qPCR) primers: primers 1 and 2 for *PAH*, primers 3 and 4 for *PAHAL*, and primers 5 and 6 for *PAHAL¯*. The length of the entire *PAH* locus is drawn to scale. The black scale bar for the *PAH* locus represents 1 kb. The gene structure of *PAHAL* and *PAHAL¯* is scaled up in blue. The blue scale bar for *PAHAL* and *PAHAL¯* represents 0.1 kb. (b) Tissue expression of *PAHAL* and *PAHAL¯* (left panel) and *PAH* (right panel) in locusts. qPCR was used for transcript quantification. The different letters within each gene indicate that the means are significantly different (*P*< 0.05). (c) The RNA levels of *PAHAL, PAHAL¯* and *PAH* in the brains of gregarious (g) and solitarious (s) locusts. Seven biological replicates of eight brains were detected. (d) Profiles of *PAHAL¯* and *PAHAL* expression in the brain during locust isolation and crowding. Seven to nine biological replicates of eight brains were measured. Asterisks indicate significant differences between each time point and 0 h (*P* < 0.05). Student’s *t* test: *P < 0.05; **P < 0.01; ***P < 0.001. (e) *PAH* and *PAHAL* mRNAs detected in locust brains using Northern blot analysis. The *U6* snRNA was used as endogenous control.

To reveal the regulatory contribution of the embedded *Gypsy* element to lncRNAs, we investigated the specific lncRNA *PAHAL*, the regulatory functions of which were well documented in our previous work [13]. In addition to the *PAHAL* transcript from the *PAH* gene, we found, using 5′ and 3′ RACE, another transcript isoform of *PAH*, hereafter named *PAHAL¯*. This transcript is 2,431 nt long and has nearly the same sequence as *PAHAL* (from +1 nt to +2395 nt) but lacks the embedded *Gypsy* element and thus can act as a control for *PAHAL* (Figure 2(a) and Supplementary Fig. S1). Similar to *PAHAL, PAHAL¯* does not possess protein-coding capacity and is a lncRNA (Supplementary Fig. S2).

We measured the expression levels of the three transcripts of the *PAH* loci, that is, *PAHAL, PAHAL¯*, and *PAH*, in five tissues in fourth-instar nymphs of G locusts (Figure 2(b)). Compared with the relatively high expression of *PAHAL* and *PAH* in the brain, the expression of *PAHAL¯* was almost undetectable (Figure 2(b)). We also analysed the expression of the three transcripts in the brains of G and S locusts since *PAH* and *PAHAL* were shown to be involved in the regulation of behavioural transition between the two phases (Figure 2(c)). The expression of *PAHAL* and *PAH* was 10.1-fold (*t* test: *P* = 0.004, N = 7) and 2.6-fold (*t* test: *P* < 0.001, N = 7) higher, respectively, in the G locusts than in the S locusts. In contrast, *PAHAL¯* presented no difference in the expression levels between the two phases (*t* test: *P* = 0.084, N = 7; Figure 2(c)).

To examine the effect of population density on the expression of *PAHAL¯* and *PAHAL*, we tested the time-course expression dynamics of *PAHAL¯* and *PAHAL* transcripts in the locust brain (Figure 2(d)). *PAHAL* was significantly upregulated at 8 h upon aggregation (*t* test: *P* = 0.005, N = 5) compared with the level at 0 h. The upregulation of *PAHAL* expression was sustained even at 32 h (*t* test: *P* < 0.0001, N = 5). In contrast, *PAHAL* expression was significantly downregulated at 4 h after isolation (*t* test: *P* < 0.0001, N = 4). This expression continued to decrease at 32 h after isolation (*t* test: *P* < 0.0001, N = 4). The time-course expression pattern of *PAHAL* is similar to that reported for *PAH* [13]. However, the expression of *PAHAL¯* was extremely low and exhibited no difference in the brains during locust aggregation and isolation. We performed Northern blot using a universal probe of the three transcripts to reveal their different transcript size and expression levels ((Figure 2(e), two biological replicates). The expression level of *PAH* is extremely higher than that of *PAHAL*. The results imply that the embedded *Gypsy* element may contribute to the regulation of lncRNA expression in response to changes in population density.

### The *Gypsy* element prolongs the half-life of *PAHAL*

**Figure 3.**
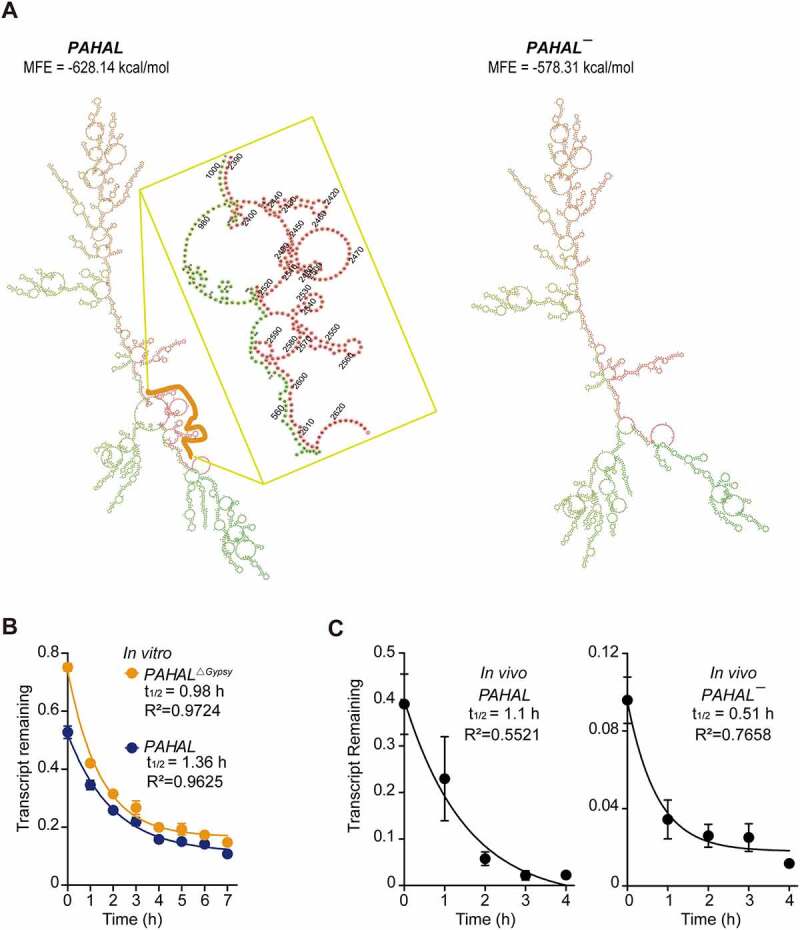
The *Gypsy* element prolongs the life-span of *PAHAL*. (a) The embedded *Gypsy* element contributes to the predicted lncRNA secondary structure. ‘MFE’ is the minimum free energy. The yellow line indicates the region of the embedded *Gypsy* element. (b) RNA stability of *PAHAL* and the *Gypsy* element-deleted *PAHAL* (i.e. *PAHAL*^Δ*Gypsy*^). The SRSF2 protein-depleted mouse embryonic fibroblast line (SRSF2-MEFs) that overexpressed *PAHAL* or *PAHAL*^Δ*Gypsy*^ was treated with the transcriptional inhibitor actinomycin D (Act D) or vehicle (0.1% DMSO) for 1 to 7 h. *PAHAL* and *PAHAL*^Δ*Gypsy*^ levels were measured using RT-qPCR (N = 7). (c) Decay of *PAHAL* and *PAHAL¯* RNA in the presence of Act D at the brain. The insets show parameters for the fitted curves using one-phase exponential decay. Eight replicates of eight brains were measured. Student’s *t* test: * P < 0.05; ** P < 0.01; *** P < 0.001; n.s., not significant.

Under the same promoter, the diverse stability of RNA may be a reason for the different abundances of *PAHAL* and *PAHAL¯* in locust brains. Therefore, we examined whether the existence of the embedded *Gypsy* element influences the stability of *PAHAL* RNA. The secondary structures of *PAHAL* predicted by RNAfold showed that the *Gypsy* element embedded in *PAHAL* has the potential to fold into a stable stem-loop structure with the other part of *PAHAL* and decrease the minimum free energy (MFE) of *PAHAL* (Figure 3(a)).

The RNA decay rate assay in the SRSF2-MEFs showed that, after transcription inhibition with actinomycin D, *PAHAL* with the deletion of *Gypsy* retroelement (labelled as *PAHAL*^Δ*Gypsy*^) showed a dramatic decrease in stability, with a short half-life of 0.98 h following transcriptional inhibition, compared to the unmodified *PAHAL*, which displayed greater stability and longer half-life of 1.36 h (Figure 3(b)). *PAHAL* RNA levels in locust brains decayed with a half-life of approximately 1.1 h. However, the RNA of *PAHAL¯* lacking the embedded *Gypsy* element displayed a half-life of only 0.51 h under the same conditions (Figure 3(c)). Therefore, the embedded *Gypsy* element helps to stabilize RNA.

### The embedded *Gypsy* element is required for nuclear retention of *PAHAL*

**Figure 4.**
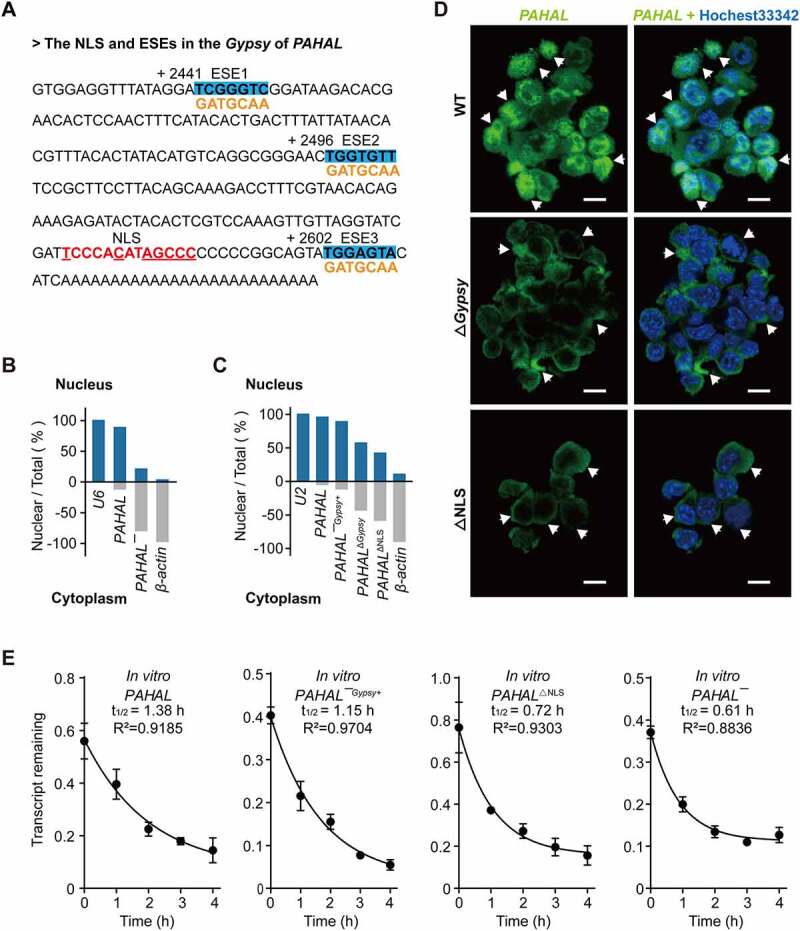
The *Gypsy* element promotes the nuclear retention of *PAHAL*. (a) The red bold text represents the nuclear location signal (NLS) sequence. The conserved nucleotides are highlighted using an underscored line. The boldface with blue shading indicates the three predicted SRSF2 high-affinity sites (ESEs) in the *Gypsy* element embedded in *PAHAL*, which are labelled ESE1, ESE2 and ESE3. ‘+2441’, ‘+2496’ and ‘+2602’ represent the start sites of the three ESEs relative to the transcript start site of *PAHAL*. The yellow characters represent the mutant sequence of the ESEs. (b) Nuclear localization of *PAHAL* and *PAHAL¯* in the locust brains. *U6* and *β-actin*, stable and abundant housekeeping genes that localize to the nucleus and cytoplasm in the locust brains, respectively, were used as internal controls to test the localization of *PAHAL* and *PAHAL¯* in the brain. Five biological replicates of twenty brains were examined for each nuclear fractionation. (c) Nucleocytoplasmic shuttling of four *PAHAL* RNA variants, i.e. wild-type *PAHAL, PAHAL¯* with insertion of the *Gypsy* element into its 3′ end (labelled as *PAHAL¯^Gypsy+^*), *PAHAL* with *Gypsy* deletion (i.e. *PAHAL*^Δ*Gypsy*^), and NLS-deleted *PAHAL* (i.e. *PAHAL*^ΔNLS^) in SRSF2-MEFs (N = 6). *U2* and *β-actin*, indicate the nucleus and cytoplasm in SRSF2-MEF, respectively, were used to determine if there were specific changes in the pattern of *PAHAL* localization elicited by *Gypsy* or NLS deletion. (d) FISH detection of the subcellular localization of *PAHAL, PAHAL*^Δ*Gypsy*^ and *PAHAL*^ΔNLS^. Images are shown at 63× magnification, and scale bars represent 10 µM. The arrows indicate the subcellular location of *PAHAL* (in the nucleus), *PAHAL*^Δ*Gypsy*^ and *PAHAL*^ΔNLS^ (in the cytoplasm). (e) The half-life of *PAHAL¯^Gypsy+^* and *PAHAL*^ΔNLS^ RNA in the presence of Act D at SRSF2-MEF. Six replicates were measured. Student’s *t* test: * P < 0.05; ** P < 0.01; *** P < 0.001; n.s., not significant.

*PAHAL* is known to localize primarily to the nucleus [13]. Sequence analysis showed that the embedded *Gypsy* element contains an NLS, indicating the modulation of the subcellular localization of *PAHAL* (Figure 4(a)). The nuclear fractionation experiment with the locust brains showed that 89% of *PAHAL* mRNA localizes in the nucleus, while only 21% of *PAHAL¯* RNA retained in the nucleus, relative to nuclear RNA *U6* (positive control) and cytoskeleton actin (negative control, Figure 4(b)). Furthermore, the intracellular distribution was quantified using nuclear fractionation of SRSF2-MEFs. Unlike *PAHAL*, which retained 96% RNA in the nucleus relative to snRNA *U2* (nuclear control) and *β-actin* (cytoplasmic control), *PAHAL*^Δ*Gypsy*^ reduced the RNA level in the nucleus to 57%. Furthermore, we deleted the NLS in *PAHAL* (*PAHAL*^ΔNLS^), which caused a reduction in RNA in the nucleus to 42% (Figure 4(c)). A rescue experiment showed that the artificial insertion of the *Gypsy* element into the 3′ end of the *PAHAL¯* (labelled as *PAHAL¯^Gypsy+^*) resulted in the nuclear retention of *PAHAL¯^Gypsy+^*, with up to 89% of *PAHAL¯^Gypsy+^* retained in the nucleus (Figure 4(c)). FISH in SRSF2-MEFs further proved that *PAHAL*^Δ*Gypsy*^ caused a pronounced reduction in the nuclear retention of *PAHAL* (Figure 4(d)). Moreover, *PAHAL*^ΔNLS^ also had a similar effect on nuclear retention (Figure 4(d)).

We tested the life time of *PAHAL*^ΔNLS^ and *PAHAL¯^Gypsy+^*. The results showed that *PAHAL¯^Gypsy+^* in the SRSF2-MEFs, upon the insertion of the *Gypsy* element, exhibited a dramatic increase in stability. However, the deletion of NLS speeded up the degradation of *PAHAL*^ΔNLS^ RNA (Figure 4(e)). Therefore, the *Gypsy* and NLS within the element confer nuclear retention of *PAHAL* and defer the degradation of *PAHAL* RNA in cytoplasm.

### The *Gypsy* element affects the recruitment of SRSF2 to *PAHAL*

**Figure 5.**
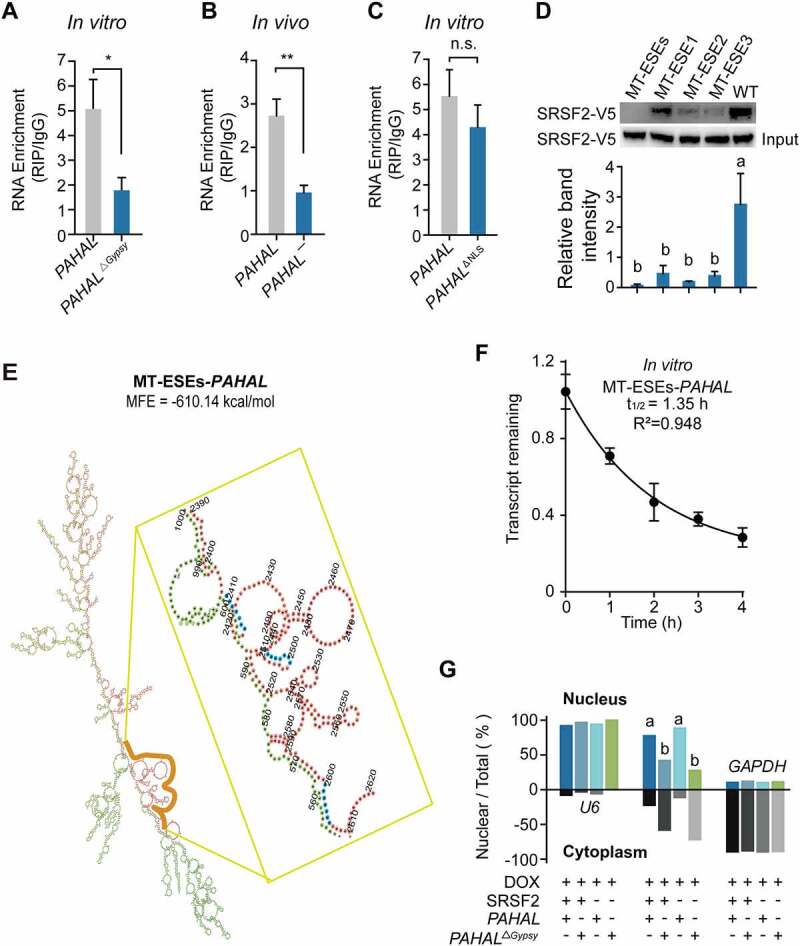
The embedded *Gypsy* element regulates the tethering of *PAHAL* with SRSF2. (a) RNA immunoprecipitation (RIP) in SRSF2-MEFs verified the effect of the embedded *Gypsy* element on the binding of SRSF2 with *PAHAL*. (b) RIP was performed in locust brains to test the binding affinity of SRSF2 to *PAHAL¯* RNA in *vivo*. Six biological replicates of fifty brains were examined. (c) Deletion of the NLS did not affect the SRSF2 binding affinity of *PAHAL*. (d) Mutational analysis of the SRSF2 affinity of the ESEs in the *Gypsy* element of *PAHAL*. Wild-type and mutant probes were incubated with SRSF2-MEF lysates that overexpressed the V5-tagged SRSF2 (SRSF2-V5) of locusts. MT-ESEs indicates the mutation of all three ESEs. MT-ESE1, MT-ESE2 or MT-ESE3 indicates mutated ESE1, ESE2 or ESE3, respectively. WT indicates wild type. (e) The predicted lncRNA secondary structure altered by mutation of ESEs in the *Gypsy* element. MT-ESEs-*PAHAL* means *PAHAL* with the mutation of ESEs in the *Gypsy* element. The blue shading indicates the ESEs of the embedded *Gypsy* element (indicated by the yellow line) in *PAHAL*. (f) The life time of MT-ESEs-*PAHAL* in the SRSF2-MEF adding Act D. (g) SRSF2 is not required for the nuclear retention of *PAHAL*. The following vectors were transfected into SRSF2-MEFs that had been treated using DOX for 1 day to knock out endogenous SRSF2: ‘SRSF2+’, pcDNA3.1/V5-His/*SRSF2* ORF; ‘SRSF2−’, pcDNA3.1/V5-His/*lacz*; ‘*PAHAL*+’, pcDNA3.1(+)/*PAHAL*; ‘*PAHAL*−’, pcDNA 3.1(+)/*lacz*; ‘*PAHAL*^Δ*Gypsy*^+’, pcDNA3.1(+)/*PAHAL*^Δ*Gypsy*^; ‘*PAHAL*^Δ*Gypsy*^−’, pcDNA 3.1(+)/*lacz*. Five replicates were measured. The different letters within each column indicate that the means are significantly different (*P* < 0.05).

Given that the most of the 3′ terminal sequence of *PAHAL* is essential for *PAHAL*–SRSF2 tethering [13], we hypothesized that the embedded *Gypsy* element is required for proper *PAHAL* binding to SRSF2. We performed *in vitro* RIP to investigate the change in the affinity between *PAHAL* and SRSF2 potentially induced by *Gypsy* deletion. Locust *SRSF2* was co-transfected with *PAHAL* or *PAHAL*^Δ*Gypsy*^ into SRSF2-MEFs in which mouse endogenous SRSF2 was depleted by adding DOX for one day. The results showed that the rate of SRSF2 enrichment by *PAHAL*^Δ*Gypsy*^ decreased by 65% compared with that by *PAHAL* (*t* test: *P* = 0.039, N = 5; Figure 5(a)). *In vivo* RIP experiment showed that *PAHAL¯* that lacks the embedded *Gypsy* element had a SRSF2 affinity similar to that of *PAHAL*^Δ*Gypsy*^ (*t* test: *P* = 0.005, N = 6; Figure 5(b)). RIP assay in SRSF2-MEFs further showed that deletion of the NLS in the *Gypsy* element didn’t affect the binding of SRSF2 with *PAHAL* (Figure 5(c)). Analysis of the *Gypsy* element sequence showed that the specific element possesses three ESEs that are required for SRSF2-*PAHAL* binding (Figure 4(a)). Next, we determined the specific sites in the *Gypsy* element involved in the interaction with SRSF2 into SRSF2-MEFs that turned off the mouse endogenous SRSF2, and then overexpressed the locust SRSF2. Mutational analysis of three ESEs within the *Gypsy* element revealed that the binding between *PAHAL* and SRSF2 was utterly disrupted by mutation of the three ESEs; in contrast, a single ESE mutation weakened the recruitment of SRSF2 (one-way ANOVA: *P* < 0.01, N = 3; Figure 5(d)). Therefore, the embedded *Gypsy* element is necessary for *PAHAL*–SRSF2 binding, and the ESEs in the element may contribute to the binding.

The secondary structures of *PAHAL* variant (i.e. MT-ESEs-*PAHAL*) with mutation of ESEs in the *Gypsy* element of *PAHAL* were predicted. The result showed that the mutation of ESEs altered the stable stem-loop structure of *PAHAL* but made no difference in the MFE (Figure 5(e)). In vitro assayed showed that the RNA half lifetime of MT-ESEs-*PAHAL* in the SRSF2-MEFs is 1.35 h, similar to that of *PAHAL* (Figure 4(e) and Figure 5(f)). Therefore, the mutation of the ESEs does not cause the rapid degradation of the mutant transcript.

We further examined whether SRSF2 affects the *Gypsy-*conferred nuclear retention of *PAHAL*. The nuclear fractionation experiment in SRSF2-MEFs demonstrated that the presence of SRSF2 did not affect the nuclear retention of *PAHAL* (Figure 5(g)). In contrast, *PAHAL*^Δ*Gypsy*^ significantly increased the nuclear export of *PAHAL* regardless of the presence of SRSF2 (Figure 5(g)). The results indicate that SRSF2 does not regulate the nuclear localization of *PAHAL* mediated by the *Gypsy* element.

### The embedded *Gypsy* element is required for transcriptional regulation of *PAHAL*

**Figure 6.**
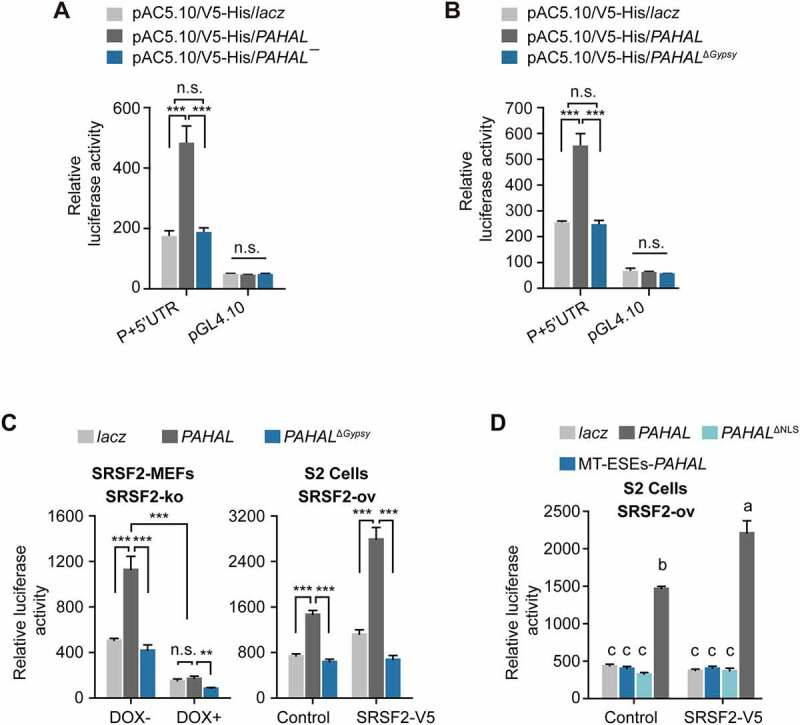
The *Gypsy* element embedded in *PAHAL* is required for *PAHAL-*mediated transcription activation. (a) *PAHAL¯* inhibits the promoter activity of *PAH*. ‘P + 5′UTR’ contains −1,168 to +89 bp relative to the TSS, representing the *PAH* promoter (‘P’: −1,168 to +1 bp) and 5′- untranslated region (‘5′UTR’: +1 to +89 bp). (b) The *Gypsy* element is required for the regulatory function of *PAHAL*. ‘*Lacz*’ is a frameshift mutational fragment of the *lacz* gene and serves as a negative control. *PAHAL, PAHAL¯, PAHAL*^Δ*Gypsy*^ and *lacz* were inserted into the pAC5.1/V5-His A vector, which is a high-level transient expression plasmid in *Drosophila* S2 cells. (c) The embedded *Gypsy* element participates in the interaction of SRSF2 and *PAHAL* for *PAH* transcription activation. SRSF2-MEFs (SRSF2-KO) were used to test the activity of the P + 5′-UTR by luciferase assays. ‘DOX +’ represents endogenic SRSF2 knockout, whereas ‘DOX – ’ or ‘Control’ indicates the normal expression of endogenic SRSF2 in SRSF2-MEFs or in S2 cells. ‘SRSF2-V5’ indicates the overexpression of the V5-tagged locust SRSF2 in S2 cells (S2, SRSF2-ov). Student’s *t* test: *P < 0.05; **P < 0.01; ***P < 0.001; n.s., nonsignificant. (d) The ESEs and NLS in the *Gypsy* of *PAHAL* contributes to the *PAHAL*-mediated transcription activation of *PAH*. Six replicates were measured. The different letters within each treatment indicate that the means are significantly different (*P* < 0.05).

We then evaluated whether the presence of the *Gypsy* element affects the regulatory function of *PAHAL*, because *PAHAL* generally promotes the transcriptional activation of the *PAH* promoter [13]. The luciferase assay in S2 cells showed that *PAHAL¯* lacking the *Gypsy* element had a 72% inhibitory effect on *PAH* promoter activity (*t* test: *P* < 0.001, N = 6; Figure 6(a)) compared with that of *PAHAL*, but its effect was similar to that of *lacz* expression (negative control). Similarly, in S2 cells, *PAHAL*^Δ*Gypsy*^ did not activate the *PAH* promoter compared with *lacz* and exhibited a 66% reduction in promoter activity compared with *PAHAL* (*t* test: *P* < 0.001, N = 5; Figure 6(b)). Therefore, the embedded *Gypsy* element is required for *PAHAL*-mediated *PAH* transcription activation.

Since *PAHAL* acts as a nuclear lncRNA to recruit SRSF2 to the *PAH* proximal promoter, we explored whether the presence of the embedded *Gypsy* element affects the interaction between *PAHAL* and SRSF2 during *PAHAL*-mediated transcriptional activation of *PAH*. Mouse endogenous SRSF2 knockout by adding DOX in SRSF2-MEFs significantly reduced *PAHAL*-mediated transcription activity (one-way ANOVA: *P* < 0.001, N = 5), and deletion of the embedded *Gypsy* element from *PAHAL* further inhibited the transcription effect by approximately 50% (one-way ANOVA: *P* = 0.001, N = 5; Figure 6(c); left panel). Moreover, while *PAHAL* with SRSF2 overexpression in S2 cells significantly boosted *PAH* promoter activity (one-way ANOVA: *P* < 0.001, N = 5; Figure 6(c); right panel), deletion of the embedded *Gypsy* element from *PAHAL* absolutely abolished the effect (one-way ANOVA: *P* < 0.001, N = 5; Figure 6(c), right panel). The *Gypsy* element and SRSF2 exhibited a significant interaction of the regulatory effects (Mann–Whitney *U* test: *P* < 0.001, N = 5; Figure 6(c)). Luciferase assay with mutation of the ESEs or NLS in *PAHAL* in S2 cells further demonstrated that the three tandem ESEs and NLS in the *Gypsy* of *PAHAL* are two elements essential for the *PAHAL*-mediated transcription activation of *PAH* (one-way ANOVA: *P* < 0.001, N = 6; Figure 6(d)). Therefore, the *Gypsy* element is crucial for the interaction of *PAHAL* and SRSF2 during transcriptional activation of *PAH* mediated by *PAHAL*.

## Discussion

In this study, we demonstrated the functional significance of a TE embedded in a lncRNA for the regulatory role of the lncRNA in the phase change of the migratory locust, because *PAHAL* is distinct as a transcriptional activator of locust behavioural plasticity, acting by accelerating ancestral *PAH* gene expression, resulting in DA production. *PAHAL* harbours a *Gypsy* element inserted at the 3′ end. The embedded *Gypsy* element is essential for *PAHAL*-mediated *PAH* transcription activation, acting by facilitating the interaction between *PAHAL* and SRSF2, promoting the nuclear retention of *PAHAL* and increasing *PAHAL* RNA stability. These findings highlight the contribution of TEs to the regulatory circuity of lncRNAs in locust phase changes. The *Gypsy* element-based *PAHAL* transcriptional regulation mechanism indicates the contribution of TEs to the regulatory circuity of lncRNAs in locust phase changes and the vital role of the embedded TEs in lncRNA-modulated protein activity. The embedded *Gypsy* element potentially provides new targets for the prevention and control of locust plagues.

**Figure 7.**
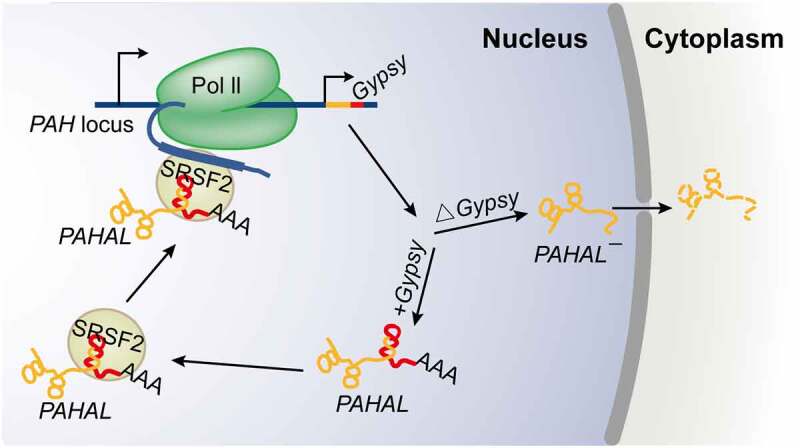
Working model for the embedded *Gypsy* element-mediated lncRNA transcriptional regulation. The *Gypsy* element embedded in *PAHAL* is required for the nuclear retention and RNA stability of *PAHAL*. Instead, the lncRNA without the element, i.e. *PAHAL¯*, is exported to the cytoplasm for decay. The embedded *Gypsy* element boosts the recruitment of SRSF2 to form an RNA-protein complex, which is crucial for the *PAHAL*-mediated promoter activation of *PAH*. The red genomic region in the *PAH* locus represents the insertion site of the *Gypsy* element.

The present study revealed the essential roles of embedded TEs in the mediation of the regulatory function of lncRNAs. Some lncRNAs are engaged in gene regulation, depending on their specific sequence and RNA structure [42]. The *Gypsy* element of *PAHAL* is an important functional region, boosting *PAH* transcription activation by not only affecting the nuclear localization and life span of *PAHAL* but also promoting the assembly of the *PAHAL*-SRSF2 regulatory complex (Figure 7). Importantly, these findings may not be limited to *PAHAL* given the presence of diverse *Gypsy* elements in numerous lncRNAs, particularly at the 3′ end of lncRNAs (Figure 1). Thus, our results indicate that a large proportion of lncRNAs are embedded with TEs, as indicated in previous studies [18,43]. All major TE classes (DNA, LTR, SINE, and LINE TEs) were detected in lncRNAs in different vertebrate species [18,44]. The TEs embedded in these lncRNAs could supply the sequences and signals involved in the transcription and processing of lncRNAs, e.g. splicing and poly(A) sites [17,18,45]. For example, the transcription start site of *LINCROR* RNA is derived from the LTR of the *HERVH* element [20]. In healthy subjects, repeat D4Z4 expansion induces transcriptional repression of the *D4Z4*-derived noncoding RNA *DBE-T* by providing PRC2 attachment sites, preventing the development of facioscapulohumeral muscular dystrophy (FSHD) [9]. The insertion of the inverted LTR of the *Gypsy* element in the *PAH* intron may provide an alternative polyadenylation processing site, which may facilitate the biogenesis of *PAHAL* and *PAHAL¯* (Figures 2(a) and 5). This raises the possibility that, although the types of TEs vary among species, the roles of these TEs in lncRNA biogenesis and regulation may be conserved.

In our study, the embedded *Gypsy* element was necessary for the nuclear retention of *PAHAL* RNA in the regulation of *PAH* transcription. Deletion of the embedded *Gypsy* element or the NLS in the *Gypsy* element resulted in the transfer of *PAHAL* RNA from the nucleus to the cytoplasm (Figure 4). Similar effects were reported in mice, in which the embedded *invSINEB2* was required for nuclear localization of the lncRNA *AS Uchl1. AS Uchl1* begins within the second intron of its target gene *Uchl1* and overlaps with the first 73 nt of the mRNA [46]. This result of intragenic lncRNAs appears different from the situation with lincRNAs, wherein lincRNAs without TEs are expressed at greater levels than lincRNAs with TEs [20]. In many cells, mRNAs containing inverted repeated *Alu* elements in their 3′UTRs are inefficiently exported to the cytoplasm [47]. Such mRNAs are retained in the nucleus through binding to paraspeckle-associated complexes [48,49].

The *Gypsy*-containing lncRNA also exhibited a more stable RNA structure and a longer lifetime of mRNA than the *Gypsy*-lacking lncRNA. The presence of the *Gypsy* element at the 3′ end of *PAHAL* may protect the lncRNA from rapid deadenylation-dependent nuclear decay by forming a triple helix RNA structure and thereby sequestering the *PAHAL* 3′ poly(A) tail within the internal loop [2,50]. Therefore, the embedded *Gypsy* element in *PAHAL* acted as a functional domain to regulate RNA export and transcription (Figure 7).

The embedded *Gypsy* element in *PAHAL* recruits the bifunctional transcription/splicing factor SRSF2 by providing three conserved and tandem ESEs to activate the TF function of SRSF2 (Figure 5). In contrast, the splicing factor function of SRSF2 is activated by binding with the lncRNA *Malat1* [51]. This suggests that although the embedded TEs are not conserved in sequence across species, they may represent conserved and discrete TF binding domains. TEs could improve the complexity of transcriptional regulation events. Embedded TEs enable a relatively small number of TFs to generate distinct combinations of TF-lncRNAs through the combined actions of lncRNAs. The tethering of hnRNPK by other lncRNAs also confirms this hypothesis. In *Xist*-mediated gene silencing, the B-repeat element of *Xist* initiates the recruitment of polycomb complexes by binding hnRNPK [52]. While maintaining nuclear speckles in normal cells, hnRNPK is recruited by *SINEB1* of *Malat1* to improve the recruitment of nuclear speckle-localized RBPs [7]. The high degree of synergy between distinct TF-lncRNA complexes is fundamental for organisms to trigger the precise spatial–temporal regulation of specific gene expression in response to a specific environmental cue. For example, the embedded *invSINEB2* element of the lncRNA *AS Uchl1* regulates *AS Uchl1* nuclear retention and consequently inhibits *AS Uchl1*-enhanced translation of the sense protein-coding *Uchl1* mRNA by recruiting IL enhancer-binding factor 3 (ILF3) [46]. The accumulation of *Alu* transcripts is responsible for age-related macular degeneration by aberrant Dicer processing [53]. In plants, TE-lncRNAs also play important roles in stress responses [54,55].

TEs could promote regulatory specificity by constructing a complex regulatory network through lncRNAs. The *Gypsy* element embedded in *PAHAL* has the potential to form a stable stem-loop structure with the other part of the lncRNA that can facilitate the recruitment of the *PAHAL*-protein regulatory complex to the specific DNA region. Similar effects were reported in other TE-embedded lncRNAs [18,56]. For example, the 7.5 tandem repeats of the A-repeats of *Xist* are necessary for X chromosome inactivation through the donation of loop secondary structures and even a tertiary architecture [57]. TE sequences can mediate hybridization to other homologous (sense or antisense) DNA or RNA sequences, for example, through RNA-DNA triplex formation [58–60]. The sense *PAHAL* lncRNA is expected to change orientation and form a triplex structure with the genomic DNA region where the *Gypsy* element is embedded [61]. Complementary interactions mediated by the embedded *Gypsy* sequences could target *PAHAL* to specific *PAH* loci [56]. *PAHAL* recruits SRSF2 via the *Gypsy* element, facilitating rapid local enrichment of SRSF2. The *PAHAL*-SRSF2 complex is brought with the promoter of *PAH* into close spatial proximity by the three-dimensional folding of chromosomes. Subsequently, the ESEs of the nascent RNA of *PAH* could compete for SRSF2 from the *PAHAL*-SRSF2 complex to activate the transcription of *PAH*. We thus speculated that the ESEs of the nascent RNA of *PAH* bind to SRSF2 with a higher affinity than the *Gypsy* element of *PAHAL*, which specifically hybridizes to *PAH* loci. Therefore, the TEs in lncRNAs appear to act as hubs where nucleic acids and proteins can be agglomerated and facilitate the regulatory specificity of lncRNAs. This mechanism is particularly important for the elaborate control of behavioural plasticity in response to changing environmental signals.

## Supplementary Material

Supplemental Material
