# Supplementary material for: A Gypsy element contributes to the nuclear retention and transcriptional regulation of the resident lncRNA in locusts
Source: RNA Biol. 2022 Jan 22;19(1):206–20. doi: 10.1080/15476286.2021.2024032 (PMC8786324; doi:10.1080/15476286.2021.2024032)
Supplement: Supplemental Material [file KRNB_A_2024032_SM3081.zip › supplementary/Supporting information Gypsy-PAHAL-RNA biology-R.docx]

**A *Gypsy* element contributes to the nuclear retention and transcriptional regulation of the resident lncRNA in locusts**

Xia Zhang ^a, c^, Ya′nan Zhu ^a, c^, Bing Chen ^d, *^ and Le Kang ^a, b, c, d, *^

^a^ State Key Laboratory of Integrated Management of Pest Insects and Rodents, Institute of Zoology, Chinese Academy of Sciences, Beijing 100101, China

^b^ Beijing Institute of Life Sciences, Chinese Academy of Sciences, Beijing 100101, China

^c^ CAS Center for Excellence in Biotic Interactions, University of Chinese Academy of Sciences, Beijing 100049, China

^d^ School of Life Sciences, Hebei University, Baoding 071002, China

^*^For correspondence: chenbing@hbu.edu.cn; [lkang@ioz.ac.cn](mailto:lkang@ioz.ac.cn)

# SUPPLEMENTARY FILES

# Supplementary Table S1. Sequences of all primers used in the study.

| Primer | Sequence (5'-3') | Notes | |  |
| --- | --- | --- | --- | --- |
| **Primer for qPCR** | | | | |
| *PAH* F | AGTACAGCCTGAGTGGAAAA | To amplify *PAH* | |  |
| *PAH* R | TGTGTGTATGGATTGTAGCG |  |  |  |
| *PAHAL* F | TATTGTGAGTCAGGATGGTG | To amplify *PAHAL* | |  |
| *PAHAL* R | ACCTAACAACTTTGGACGAG |  |  |  |
| *PAHAL¯* F | GTGCAAATTGCCCCACAGAT | To amplify *PAHAL¯* | |  |
| *PAHAL¯* R | TTCATCAGGAAAGAACATACGT |  |  |  |
| *RP49* F | CGTAAACCGAAGGGAATTGA | To amplify locust *RP49* | |  |
| *RP49* R | GAAGAAACTGCATGGGCAAT |  |  |  |
| *β-actin* F | AATTACCATTGGTAACGAGCGATT | To amplify mouse *β-actin* | |  |
| *β-actin* R | TGCTTCCATACCCAGGAATGA |  |  |  |
| *U2* F | CCTGGCGTAAAGGTGGTG | To amplify mouse *U2* | |  |
| *U2* R | CGTGGAGTGGACGGAGCA |  |  |  |
| *U6* F | GAACGATACAGAGAAGATTAG | To amplify of locust *U6* | |  |
| *U6* R | AAATGTGGAACGCTTCACG |  |  |  |
| *β-actin* F | AATTACCATTGGTAACGAGCGATT | To amplify locust *β-actin* | |  |
| *β-actin* R | TGCTTCCATACCCAGGAATGA |  |  |  |
| **Primer for FISH** | | | | |
| *PAHAL* F1 | CTGCCTGCTTCTTGTCATG | To amplify the DNA template in *PAHAL* RNA probe 1 synthesis | |  |
| *PAHAL* R1 | GACAGGTTGTCTTGCGATC |  |  |  |
| *PAHAL* F2 | AAGGTTATACTAGTATCTATTTC | To amplify the DNA template in *PAHAL* RNA probe 2 synthesis | |  |
| *PAHAL* R2 | ACCTTCCATTGATAGAAACAG |  |  |  |
| *PAHAL* F3 | CTGCCTGCTTCTTGTCATG | To amplify DNA template in *PAHAL* RNA probe 3 synthesis | |  |
| *PAHAL* R3 | ACCTTCCATTGATAGAAACAG |  |  |  |
| **Primer for Clone** | | | | |
| *PAHAL* F | GGTAAGCAGCACATTTTGTACA | To amplify full-length *PAHAL* | |  |
| *PAHAL* R | GATGTACTCCATACTGCCGG |  |  |  |
| *PAHAL¯* R | GGCATAACCTTCATCAGGAAAG | To amplify full-length *PAHAL¯* using *PAHAL* F and *PAHAL¯* R | |  |
| *PAHAL*^Δ^*^Gypsy^* R | CACAATCATACAGGAACAACTC | To amplify full-length *PAHAL*^Δ^*^Gypsy^* using *PAHAL* F and *PAHAL*^Δ^*^Gypsy^* R | |  |
| **Primer for Vector Construction** | | | | |
| *PAHAL* KpnI F | GCGGTACCGGTAAGCAGCACATTTTGTACA | To amplify *PAHAL¯* with *PAHAL* KpnI F and *PAHAL¯* XhoI R | |  |
| *PAHAL¯* XhoI R | GCCTCGAGGGCATAACCTTCATCAGGAAAG |  |  |  |
| *PAHAL*^Δ^*^Gypsy^* xhoI R | GCCTCGAGCACAATCATACAGGAACAACTC | To amplify *PAHAL*^Δ^*^Gypsy^* with *PAHAL* KpnI F and *PAHAL*^Δ^*^Gypsy^* xhoI R | |  |
| *PAHAL*^ΔNLS^ xhoI R | GCCTCGAGTCGATACCTAACAACTTTGGAC | To amplify *PAHAL*^ΔNLS^ with *PAHAL* KpnI F and *PAHAL*^ΔNLS^ xhoI R | |  |
| *PAHAL¯* F for *PAHAL¯^Gypsy+^*pcDNA | AGCGTTTAAACTTAAGCTTGGGTAAGCAGCACATTTTGTACA | To amplify *PAHAL¯* for two fragments insertion into pcDNA3.1(+) by In-Fusion cloning | |  |
| *PAHAL¯* R for *PAHAL¯^Gypsy+^*pcDNA | AACCTCCACAGGGCATAACCTTCATCAGGAAAGA |  |  |  |
| *Gypsy* F for *PAHAL¯^Gypsy+^*pcDNA | GGTTATGCCCTGTGGAGGTTTATAGGATCGGG | To amplify *Gypsy* for two fragments insertion into pcDNA3.1(+) by In-Fusion cloning | |  |
| *Gypsy* R for *PAHAL¯^Gypsy+^*pcDNA | GACTAGTGGATCCGAGCTCGGATGTACTCCATACTGCCGGGG |  |  |  |
| **Primer for Northern blot analysis** | | |  | |
| *PAHAL-PAH* F | ATTGGTCTAGCCTCCCTTG | To amplify the DNA template in *PAHAL-PAH* RNA probe synthesis | |  |
| *PAHAL-PAH* R | TTGTGCCTCTATGCTGCTC |  |  |  |

**> Full-length sequence of *PAH* locus**

**GACAGCGCGGGGCCAGAGCAGGTGGCGAGCCGAGGAAGCGGGCAGACGAGATCGGCAGGCGCAGCGACTACACACGCAACGCAACACAATGGGACTCTCTGAGCTGCCGCCGTCGCCCGAAGAGATCGTCGAAGAC**GTAAGTTAATACTTCACCAAGTGCTAGGCCGACCAACACGTGATCGTAATTTTAGGGAGATGGCCGACCGTGATTTTAACCCTTTAATTCATGACTTTTTATTTGAAGCTAAAAAATACCACGATTAATGTATAGCGCGTATATACTGAGAAAAATTTTGAAAAAAAAATCCTTAAAATTATTATGCACCACTAGCAACAAAAAGTTTATAACTGGTCAGCTATACAGTGCTTCTGTGCTGTACCCGATGTAGTAAATGTCACTATTATGCCTCTCATATTTCCGGAAAGTAAGTCATAATGCAACAGCAGTATGTGATTGACTACTTTTACATGGCAAAGTAGGAATACTATTCTAAACAAACGAACATGTTAGGAGTAACTCAATGCAACTCACTGTCGAAATGCTTCAGTAACAGTCTGCAATTTATAATTTATAGGACGAGCTACTAGCATGTATAACACTATGTATACAAGCAGCTATAAATGAATTTGTGTTAAAATTTTGAACAGTTTGAGAGATAATGGGAAAAATTAAGTATGTACCTGATCAGCTACACTATGCATTGAAGGGTTAAGGAAGGGGTGACATAATCGCACCAAAAAAGGAGGAACAGGGGTAGCCTAATGACGCCACCTTAACGGAAACCGGAATGTTTTCGCCGTATTAAAAGTGTATCTTTTTCTTACAAAATAATACTATTCTTGTTATGGTGGTTGTCTTCAGTCGAAAGACTGGTTTCTTGCGGCTCTCTACACTACCCTTTCCTGCACAAAAACCTTTATCTCCGAATAAGAAAGGAAACCCACATCCTTTTGAACTTCGCCACTTACCTTCTCTCTTTGCGATTTTTACACCAATGCTAAATTGATAATTCTTTGATATCTCAGAATGTATCGCACCAAGCTATCACTTCTTGTAGTCAAGATATGTCTTAAATTTCTTTCCTCCCCCGTATTTTGTTCAGTGTCTCCTCATTAGTTACTCGATATAATCGTCTAATCTTCATAATTCTACTGTAGCACCCCATCTTTTTCTCTGAACTCATCGTCCACGTTTCACTCCTGATCATTTCCGACGGAACGTGTGGTGCAGTGGTTAGCATCACAGATCGCTGTGTTGTGGTCCCCAGTTCAAATCCTACCACTGGCAGAAGTAATTATTTGTTTGTATTTATTATTTCTCGAAGTTCTACATGTTTGTCATGTTTGATGTACTTGTAAGTTCCAGAAAATTCTTTATATGTATAAACAGTGGAATACTCCAAGCAGGAGTCAGTCTGTAGTGAGGATACGCTTGTGTTAGCTATAGACGTGATCTAAGTGTAAAGTGTTGTGCTATGTTAACCACCAGCCTTCGAATTCTAGACGAGCTGCAGTTTGCAGCGTGATAATATTACACTCCAGACAAACACTTTCAGAAAGGACTTCCTAACATTTAAATTAATATTTGACGTCAACAAATTTCTCTCCTTCGACAATGCTTTTCTTCGCATAGCCATTCTCTATTCTATATCTTCTTTATTTCGACCACCAGAAGTTATTTTATTATGCAAATAACAAAACTCGTCTACTTCTTTTATTGTCTCGTTTCCTAATCTGATTCCTTCATAATCGACTAATTTTTCTTAGAATTCATCCCATTCCCTTTGTTTTACACTTGTTAATGTTCGTCTGATATCCTACTTTGAAGACACTATCCATTCCATAATACTGTAGCTTCACCGATTATTACAACTTTGTTATAATTGTGTTCAGCCCTCTGCCTTTCTTTTTTTTGCGTTTTATTGTTAAAGATACGTCGTGAAAGTAGCTCTAGTGGCGCTGTTACCCTGGTTTCTGGTATAATAACGCGTTTTCCATGGGGTAAATACAGCATACTTTTAAAAATTCAAAAAAGTAGCCTGCATATTGACAGACTACTAGTAAAAAAGTATTGCAACTGTTGTAATTTAGACAAGTGAAAAGGTGGGGGTTAACAGCGTTGATATATTTGTAGGATGACATTATAATTGACCACAGCTGTTTATTTAATGACCCAATGTTACCGGTTTCGGTTTTACATCATTTTCAAGCGCCAGCAGAGCTTAAACTTTTGATTAAAATGACATGTTACAATCTTGAAAATGAAAAAGTATAAAATAGATATCTCGCATTCTACTGGAATTGCTAGATGTGTATCTCAAGAGACTGCTCATTACTTGTAATTTACGCACATCATTCTTTTCAGTGTGACAGTAAGAAGGCGTAATTATACTTTCGACAGTGAGACAGTAAGAAGGCGTAATTATACTTTCGATTAAAACTGGAATAAAGATAAATGTTATAAGTTATTCCTGTGATATCTTAAATATAGTTTTAACTCTACATATTTGCCATAATATCTACAGCTGTACCACAGATCATTTTGTCCACATTCTCCACAAATTTCCACACTGCTTTACGCACTCTTTCATAAGATTTTGGAAAGAGATTCAGTAATTCAGAAGGTTCATGCGCAATCTCACCTCGTTTACATTGTTCCTGCATTTGAACTCACCTCACCTTACCAGAGATTGACAAAACAAACACTACGGTGCAGCTGTCGCAAATCAGTAAGAGACTTGAGAACATAAATAATTTACTAATGAGCTACGCACTTTATCCCTTGTATTACGCCAAACAGGGCATGATTCTGTAGAGAAAGTAATTCCATGCCAAACCTTTTGAGTTTGTTCAAAGTCGTCACCCGTCGACTGCTTGTCATTACTTTCCCGGTCTCGTGTAGGCGATGTAATTCTAAGCTTGCGGCGTGCTTTCTGTTACAG**CCGATGCTGATGACGGGAGGCAACTACATCAAGGAGGGCCTCGACTCAGCCAAGAGCATTTGCCTCATCTTCTCGCCGCGAGACGGGGACCAGGTCGGCGGACTCGCCAAATGCCTCAAGCTCTTCGAG**GTGAGTCACTCTCCAGTTCTTCGTTATTCATATAATTATCCTGGGTTGGTGTCCACATAGTATTGTAAACCTAACAACTGGTTCAATTACTATTACAAGTGATCTTCATTGGGTTTGTCACATAATCTAAATATATATACACGAATGTTCGTCTGTATGTCCTCTATGTGTTCATAAACCATTCATTCGTTTGCAATGAAACTTTGGTGAACTGTTCACCGTGCGCCCACGAAGATTCCTGATTCAAAAACAAGAGAGCACGACACATAGCTCAGGAGATATGACGTCATAAATAATGAGAGGCCACCGCGAGAGAATACGCAGATTTATGCCTCAAAATTGGAAAATGAGAGTAGTTAGGGACCTGCAACAAGGTTTGCATGTAATTTCGGACGTTTATGAAGCGTTTTATAACCGACACCACACACACACACACACACACACACACAATGATGAAACGATAAGAAGTCTACCGCTCACAACAGTTTCGCTGTAGATGCTGTAAAATTGTCGCATCAAATAAAGTTAGTATTACAGATTTGCATGTTACTATTTCTTTTTGTCTGCAAGTCTCCCTTAATTCCCTTGCATCCAAAATTAAGCAATCTGTTTTCTTAATCTCCATCTATAGAAAAGCGAAAGGACACTCACTCACTGACTAAACACGAAATCTAGAAAACTACTTCACCTATGAAGCTGGAATTTGGCAGGAATGTAGTTTATAGGTAGAAAAATCAGCTAAGAAAGTATTTTGTGATAGGTGCATGGCGGAGCATACTTCTCCTACAGACTCAAATCTACAGTTTCATTCCGATCTTCACGAAATTCCGCACACTTGATCTTCAAAACAAGGGGTAGATCACCTTCTGCCTTAAGTTTCATACGGTGCATGGAGGAGGGTACTTTGCAACATAAAAATGGATCCCTAAATACCTTAACTTTAGAACGGTTCGAGCGATTTCGCAGATACTTTTTTTTTTTTTTTTTTTTTTTTTTTTTTTTTTTNNAACAAGGGTTGTATGAAATAAACCTGGAATATTTGAGAACAACAAAGTGCGTTTTTCAGTGTGACCCTGAGTTTCTATTGCATGGAAATCTAAAGCTTTGTTTCGATATTCCTGAAATTTTGATCACTCCACCCTTTTTTTTTAAAAAAATAAATAAATAAAAATAAAAAAAAACTAGTGGAATGCCACTGTCTGTATAAAATTTACTTAACTTACGGAATTACACAGAGTGCATATTTTTGCTCCAATCTTTATGAATTTTTGCATACATGACCTTTAAACCAACAGGAAGATCACTGGGTATATAAATGTTTGTATGGTACATGACAGAGGGTACGTTGCAGAAAACTTTGCCGAGAGAGAGAAAGGTGGAAACGAACAGAGAGAAGGGAACTGGACAAATTGAAGGAACCAGAGTTGGTTGAGGATTTCAGAGGGAGCGGGGAGGGGGGTGTAGGATAAGTAAGCACTCGGGCAAAACCGGGTTTACATGTAAAACTTCTCTAAATTGCTTTACACCAACCTATTACGCGGCAGTGAAACTGAGAAGTTGCTTACGACTAACTTGAAAGCTCTGCGGAGACACACACGACAGATATGTCAAAAGCCTTCACTTAAGCTTCTTTGCTTTCTTTGTCCCTACGCAACAGAATGTCATCGACCTTCGAGACAATGCCAGTGATACTTACCGCTCACATGAAAACGGCTTATGAGTAGGATACATAAATTGAATAGCTAATATTTTGTTTACCTATCTTTCCCCATTACGGAATTGGAGGTTTCTAAGGCGTCTGCAATAGCTACGATAACTTATACTAAATTAGACTTACTCTTCTTTATCAGTCACCACTTAAAGAAAAGATTAGCATAATGTATGCTTGTTGTTCTGGTGTTATATTAGCGATTGACACCTTAGTAGCAATAGCCAGTCACTCTTCCGCCATTCTGCTAAATCTCCACTGTGTTATATCGGCACGCGAAAACATGTTTGCTTACTTTCACTCTGTCAGGTTCTACGGGTCAGTTGTAGTTGTGTGCTTTCCTTGGACAGTGAGGGCTAATAGAACGTCTGCACTGTAATTTGGTATACTTTGTATAAGACACTGTCCAAGGGTCTAGTAGCAATTATTCCACTGCTATCTCAGTGCATATACTTTCTCATGAGGCTTTCACTTATTAACAATGTCTCAACTGATAGTAACGTTACATGCACTACGCGAACAGTAGACGAAAAAAAAAGCACTTATACGACTGCTTCTTAACAATTGTTCGGATCAGTGTACACTACTATGCAAGATTCATTTACAACGACACTCCAACTTAAGTACAAAGAGAGCGATATAAACAGGGTCGATGTGTTTGTTACACATTTCCTACATTATTCCAGGCTTTCTTTCTATAGTATAAAACATTCCGGTGTCATGCGGTATCTTATACATTTTCTCTAACAAATATATCTCTTTTAAATCAATATTCCTTGTACGCCTGTATACAGGGTGACTCACTTAAACTTTTCATCCCAAATACCTCTGAAACAACAATGGATATTGAAAGACGACTTCCGACCATGAACGTACGGCAGGGATAATGAAAGTAAGTACTATGAAACATTCTAAAACGTGTAGAAATATTATTTTCAAAGCAAATATATGTGTTCTTAAATGGACACCGCATATTATTTTTCATGTAGTCAATAGCATGAATGGTGGTGGTCGCATCGCAATACGTCATTTACGTGCCGAGAAATTGGGAAGCGAATTTGAGTCTTCAAATAAATGATGCGCGTAGACAGCACAGCGCACCATTAAGGTGAGGTTCACGATTGAGTTTCGGGACGTGCGGTAGCTGAGCGTCTCGTTTATTTCAAGCGTCAAATTACAATTTCTCGGGATGTAACTGACGTATTGGGATGGCGCCGCCCGCTTTTCTGATTTCTGGCTTTGCGCAGAGATTTACTACAGAGTAGTATTAACCATTACGGCTTGGCCTACTAATTCGAGTTATTATTGTGTGTGGGATTTGGACCACTGTATTGAATCGTGTCTGCTGGTTCAGGGAAAGTGGATGGTGAATAATTCTGGGTTACCCTATGTTGTAGACAACAGAGTAACCGACCGCTTATAGGGGTCGTCTGGTTCGTTTGGTTGACAAAGTTGCCTGGTTAGAGGATTCGTGGAATCCCGCGTGCGTGCATTGAACTTTCGTGCTATTTTTTGGAACCTCTCCAGGATGTCTTTCTTTTGTGCAGCTTCTTGTAGGTACTGGTGTGAGAAGTCAGACGCTACGTGAAACACACGGCGAAGTATCTTATTTTGTTAACTTTGCTGTCTCCTCATGGGGTGTCCATTAAAAAAATATATTTAAAAAAAATTAAAAAACATAAGTTTGTGTTGAAAATAATTTTTGTTTACTTTCTAGAATGTCTCATAGTATTTGCTTTCATAATTTCGAAGTCTGTTAGTTCCCGTCGTGCCGCCACAATGAAGTCAGAAATCTTTCCATGTGGTTTAGCAGGATACAAATGACGGCTTTTACACCTTACGGACGCGATATTACCGCAGTCTGTATCTGACAAGGAGAGGACGCCTACTCGTCGTCGCCGAATTCGTCCAAATTAATGATACATGTAGGGCATGGGGAGATATGAAAGTGCACCAAGTGAGAACTCCAGATGGCTAAGCGTTTAGGAAATAAAAGGAAGTGTGATACGGATCTATCACCATATGCATCTTATCAACTGTCCCTGTGACGGTGTTTGGCTCAGCGGTAAGAGATCGAATTCGAGTTGAGAAGGTCACGGGCTTAACTCCACCCCGCGGCAATTTTTTTCTGCTCCTTGTCAATGCGTACACCTACATATTGTTTCCGTAGGGATCGTGAGTACAAGATTAGATTAATTACAGCACGCACGGAGGCATTTAAACAATCATTCTTCCCACGCTCTATACGTGAATTGAATGGGGAAAACCCTAATAACTGGTACAGCGGGACATATCCTCTGCCATGCACTTCACAGTGGTTTGCAGAGTATTGATGTAGATGTAAACTGACGTCACAATTAACCGCAACATTTTTTTCTGATGAATCGAGGTTCTGTTTACAGCATCATGATGGTCGCACCCGTGTTTGGCGACATCGCGGTGAACGCACATGGGAAGCGGATTCGTCATAGCCATACTGGCGTATCACCCGGCTTGATGGTATGGGGTGCCATTGGTTACAAGTCTCGGTCACCTCTTGTTCGCATCGACGGCACTTTGAACAGTCGCCGTTACAGTTCAGATGTGTTACGACCCGTGGCTCTACCCTTCATTCGATCCCTGCGAAACCCTACATTCACTTCATCAGGATAATGCACGACCGCATATTGCAGGTCCAGTACGGGCCTTTCTGGAATACGGAAATGTTCGACTGCTGCCCTAGCCAGCACATTCTCCAGATCAATTGAAAACGTCTGGTCAATGGTGGCCGAGCAACTGGCTCGCCACAATACGCCAGTCACTGCTCTTAATGAACTGTGGTATCGTGTTGAAGATGCATGGGCATCTAAACCTGTACAAACCATCCACGTTCTGTTTGACTCAATGCCGTTATTACGGCCAGAGTTGGCTGATCTGGGTACTGATTTCTCAGAATCTATGGACCCAAATTGCGTGAAAATGTAATCACATGTCTGTTCTAGTGTAATATATATACGTCCAGTGAATACCCGTTTATCATCCTCATTTCTTCTTGGTGTAGCAATTTTAATGGCGAGTAGTGTAGATTGGTGTGGCGTCTGTTCTTTCGAACAAGTCCGAAGAGGGCGTTTGTTTCATTTACTTCAGCGCAAAGACCTCGCACGGGAATCCATAATGGGAAGAGTAAGGCTGGTTGCACACACACCGAGTTTGGACGGCCGGTAAATATCGGGCGGTATTTTACCGGTGGAGCGCTTCTTGCACAGACGCCGGCATTTCACCGGTCAAACGAGCAGTCGCTTGGCTGTCGTGGTCGACTGAAGCCCAAGAGGGCATGGACAATGGTGGAAGTTTGAGACGGACGTTAGGCGACGCGGGTAGCCAACTGGTGCCGGCGTTCTCTGTAATAAAAAAACTGAGCTAAAAACTCTACCAACAACCCAGAACGAACACGAACAATCGACCGCAAAGAATCGATTTACCTGAGGGGGAAAAAATCGTTAAGGCGACCGCTTGCTCGAAGCGACAGACCCGGGCTCGATTACCGACACGACTCACGTTTTCACTCGTCGTCATTGTATAATGTCAGTGCCCCAAAGCTAAAAGTTAATCCTTTCCTTCCTAATCCTTCTTCCTTCCCCTCCCTAATAAATAATTTGTAAAGGTAATAATTTCATAGCAGTAGCAATTAGAAATACTAAAGCAATTAGCACAGAAATACAAATAATTTGACGTTTTCGTTGTGGGTTTAAGCCAAATGAGAAGTCCAAGCTCCAAGCGCAGCTCCAGTAATACTGAAAGGGGAACGGATGTGAAATATGATAATTTACTGCTATATTTCTTCTGTAAATCTGTGCTATTTGGAAGTTATTGAAGTTATTGAAGCACTGATAACAAAGGCCACTTGGCAAAGGTTTATTATTTTTCGATTTTTTAAAAATTACTTTATATTCATACTTACATATTTCGATGAAATTTTTCGTAAATAGAAATATATTACTCAGTCATATGGGCTTAATATTCTTTGAAGACTGATTAATATACGTAAACCTCTGGCAAAGAATACAAAGACAATGAAACGTTCATTTTTTCCGAACTATAAAGACAGGAGATGGGTCTCCATTTACTTCTTGTTCGAACATTGAGACAGTAAAGTTGTAAATGATTTGAGCCCTGTAGTTATTACGATTTTTCCAGAATATGTACAATCTATGTAAGTTATTTAAGTTTTATGTAGATATGCTACTTTTTAATTCAATTATAAGCATTTTAGTTTAACCGAACTTTTAATGTGTTCTCATTTAGTAGAAAACTGATTCCTTAATTTTTTTCCTTCTCACATTTTATTAACTGTGATGCAATTTACTTTTCAGGACAACGAATTCCGCTATTACATCCAATTACGAAATTAATTGTTGGAGGTGTGATCAGAATAGTAATCATTTTCATTCCATTCTTGTACTAAATAATTGTTCTGAATACGATCCTGAGATTTTGTGCCGAGTTATTAAATTTCAGTTTTAAAAATCTGAGCTACCCGTGGACTTTGTTAGTAAATGATAGTAAATGACGTCTCCAGCAGATAGGTTTATGGCGGTTTTCCATCAACAAAAGGTTTTTAATTTATTTCTTAGTGCGTATCTCGCATTTTCTTTAAAAATGCTGGATTTTTTCAATCTTTTAAAAATAGAGGCACAGTTTCATGACTCACATAAATGCTACGACACCAATTTGAAATATTTTTCCCGTTCAATCATTATAGCACCGTTAGTGATAGCTTTTTGAAAAATATAACCTGGCTTTTACAGTGATTTCGTTCAAATCAATATTTCCTTCAATGTTTTCATTTATTAGAATCTCAGAACTGTTTCATTTGCAGGAGCTACTCCACTTCAAGAATATTTGTAAACATATATCCAATATATAAAGCAGAATGACCGTATGTTTGTGTGTACGTGTTGCATGAAAATCTATAGTTTTGTTCCAATCTTTATCAAATTTTGTACACGCAAACTTCAATCCATGACGACGGTCGCCGTAAGTGTAAGACATATGACGTCATAAACAATGTGATGCGCAAAAACCTGCTGCATTTAGCGACCATGCTGCTCATTTTCTCCTAAACCACTGGGCCGATTTCAACCAAATTTGGTACACACATTCCTTACCGTGAGGTAACAATTGCTGCAGGGTTAAGAATCACATACAACACATGGTTCAGAACTTATGACGTCATAAATATAGGAATCGCAAAAAAGTGTCACATTATGCGGCGTGACTTTATAATTACGCTAAGAATGAGAGTACTTTGCGACTTGCAACAAACTTTAAACATTAATTCAAACCTTTCTTAACCTTTTCTCACTGACAACCATAGCAACCATAGCAAAATGGACTATAACTACCGCATTCGGTATGACGTTACAAAGTATAATTTGCACACTACAAATTGTAGCCGCAGGAAATTTTACAAACAGTATACAAGCATATAGTTGAGTGTACCTTCTAAATTGTATCACAGTACGATACGTAGTTCATGATATATGAGTACATAAACAGTAGCGTTCATAGAAAATCGCCCAATCACGCACCAATTTTTAATCTATTACTTCTTTACTACTAATTGTATTCCCAAGAGAATTTACATAAAGCTCTTGTACGAATGAATATATACCTGAAAAAAGGCAGGAATAAATAAATAAATACCCGGGCAACGCTGGGATTTAGCTAGTTAATTAAATAATAGTCATACCTAAAAATTTTAACAATAGGGGAGAAATGTTTCATTTTGGCGCCCAGCTTGGGGCTCGAACCCACGCTCTGAGATAAATAGTCTCATACTCACCGACTGAGTCGGGCCTTCCCAGTCCCCTGCCGAGAATCGAACCCGGCCTCCCTGCGTCTCGTGGACGACGAAGCAGTAGATATGTAGCGCCAATTCGCTTTGTTCGAGGAAGAAAAATGAATTATTCCGGTATTTACCTTGAGTGGTTAAAGAAAAATCACGGGAAACCTTAACGAGGGTAATACGGCGAAGATTTAAACTACTATTTTCCCGAGCGCATGACCAGCCTATTAATCACTGCTTTTCCTAGCTCGGTTCTGCAAAGAATTCTACTGTCTTAACTACTGCACCGCCTTCACGAACGTGTTTCAATGAGAAGCATGACAACAGTTCACGACTGCGCAGATATCCCCGGAAGGCTGTCCAGAGTGACGCACACATGCGTTGCAATAGGACTGTGACTTTCTCCGACTGCCAGAGATTCCAACAAGAGAGCGCTCACGCAGGGAAAACTTGGGTTTTCACGGCGTGGTTGTTCTTCCTGGTGTGCGCACAGACTGGAGACCCCATAACGCCAGCTAATTGCTCTCGTTCAGTGGCGTAACTATAGAGGGGGGGATGAGGGGGATAGAACCCCCCCTCCCAGAAACCGGAAAAGAAAGAAAGAAAGAAAGAAAGAAATCGTGCCGGTCGCGGTGGCCGAGGGGACTGAGGCGCTCCAGTCACGGTCTGCACGGCCACTACGGTCGGGGGTTCGAATCCCCTCTCGGGCATGGATATTTGTGCCGTCCTTAGTATAAGGTAGTTGATAAGTAGATTGTAAGTTTAGGGACTGATGACCCTGTAGTTTAGTCCCATAAGCTTAAAAAAAAAAATAAAAAAATCGTATAACAGCAGTATAGATACTCTGCAAATCACATTAAAGTGCCTGGCAGAGGGTTCATTACCCACTTTCATAACTTTTCTCTACCGTTCCATTCTCTAATAGCGCGCGGGAAGAAAGAACACCTAAATCGTTCCGTTTTCGATCTGATTTCTCTTATTTTATTGGGATGGCCATGTCTCCCTAAGTAGGTGGGTGCTAGCAAAATATTTTCACATTCAGAACTAAAAGTCGATGATTGAAACTTCGCAAACAGATCACGCCGCAATGAATAACGTCTTTCTTTTAATGATTTCCACCCCAATTCGTGCAACATTTCCGTGACGCTTTCCCCCCCGTAACGTGATAAAACAAAACGAGCTGCCCTTCTCTGAACCTTCTCAATATCTTCCGTCAGTCCCACCTGGTGAGGGCTTTGGATGGGAGTGAATTAGTTTCTTCAACGGGAGTGGTAGATAGTAGTCCGTCTGGAAAGTGACTAAAATTTTGCTCGTCAAGCAGATCTATCCTCCCCCCCCAAAAGCCAAGTCCTAGTTACGCCACTGTTCTCGTTGCTATAGGATTGACTGTACCTAGCAGCAACGGCACGTTGAATAGATTGTAAACATAGTTTACAGAAGGCGTGATGTAGTTTTACTAAATACGTCATCTCCTAACAAACTTTACAGCCGATGTGCCAATTTCCAGCGGGAGCTCACATTCCGGAAAATCTACTGCAGAACTGAATCCTCTATTGTATATTTATTTCCTCAACCTCTTCAATGATTCTTATATCGTGTGCTATCAAGTTTAGAATATGAATTTCGAGAGGCTATGCATACTCCTAATTCCTAATAATCACTGAGATAACTTTATGGTCCTGTTAGAAAGATCGCCGCAATATTGTATTTAAATCTACTACTTAAAAACGATCAATGTAACATTCTTTACTTGTTGACAAATCCACTTCAAAATACGCCCCTCCACGTTTCTTACACCGTTCCATACGAATTTCCTATTGCTCATACTGCCAAATTTGAAGTTATCTTCTGAAACCATCTCCATTGTCTCCGTCACTTTTTTTCACGGATTCAGCTGTTTAAAAACGTCCTCCCTTGAGTGCAGACTTCACTTTCGGTAACTGAAAAAAGTCATGCAGAGCAAGATATTCGAATAGGGTGGATGTTCCAGAACAGGTATCCCATGCATACTCAAAACCGACTTGAGAGGCGTTCGGATCTTTGTAATTATTTTTTAATAAACATTCAGAAGTGAAAATGTGAATTTAATATCTACCTGATTTTTTTCCTATTTTTGACAATTCTTTCATTACCGGCATCCACATGAATACTTCGGTTTCTTCAGTGTCTTCCACCGTTGTTGACGTTGAAGGCGCGCTAAACTAAATTAATTCATCGTCAGTCTCTTCTAGTACTTCTTTAAACTGTGAAAACCACTCACTGTCTCTAATGACCAAGCTGTCGACAGGACGTTAAAGCTTCCTTTCTTTTTTTGCTTAAACCATTCAAAAACTTCATGTTAGTAATTCATCACATCATTGTTTCAATAAATAATGGGCATCTGTTGCTATCTTTCACATTTTCATACAAAATATCTCATTATTTGTTGCTCTTACGTAGTTTTTTACCCTATGGTCTGCCGACTTCTCAGTCGGACCGTGGTCTTTGTGTATGTGAAGGTTTCTCCTTGAGGGCCGCGCTGTAGTAGGACCTCATGTTTTCTGCAAGCCTATGACGTCTGGCAAAGGGAAAACTGGCAGCTGCTTAACCTCTGTGTGTGCTCCATCTCTTTAACTTCACTTTTATAGTCTTCTCGTGAGGCTTCAGTTGAAGGAAGTACTTGAGGTCTTCTCGTGAGTTATAAATTGATGAAAGTAATTTACTGGTTGGCTCTTCTGGAAATGTAAGCTCTCGAGATTTTGAAAATAATCAATGACGACTTTGTTCCTTCAAACTGTTATTCCATGCTCTCATTTGTGTGTAACTGTGATAATTAGTCGACTCAGTGGCACTATTGATGTGAAGTCATAATTATGTAATAAAATTTCACAATAAGGTTATTTATTATAGACTGACAAATATACTTTCAGACAGTCTCTTCTATTAACATCGGGCGACGATGACGCAAGCGAGTAGATACTTCAACCTATCATGCAACTCAGCAAAGCAAAACAATATTTGCTCTACAATCGCGTTAGAAACGTCGCAATAAATCAGTCCACATGGGTAGATTACTCAATTTCACGAGATATGGGTGAAGAAAAAAAAAAGTAAGTAAAACTCTTTCAGTTAATGCGACAGTTTGCGTCTCGGGGTGTACCGGTATTCTTTATTAGGACCAGTTCTTACTATCACAGCTAATATCAGCTTATTTCGCTGTTTTTGAGTATTTAATCAAGGTAAGGTGATGGAATCATAGAAATGACAGCGAAGTCACTCGATGTGCTACCATTCTTGGTTCTTTTAAAACTTAAAACTATGCTACAAAATTTTAAACCTGTCTTTATCTATTTTGGTTTTAAGTGTACTGACTGTTTTAAACCATCTATGGAGATTTCACTACCTTAAAAGGCAGCGACTGTTTGTAAACTATCGTACCGGAACTGGAACATTCCTAACGTACGAGGGCGTGTTGGAAGGTTTATGTGAAAACTCTTCAGGCATTTTCTGCGTGCCTATTTCTTTCCCAACGTAGTCATCCTAATAAAGAATCACAGTTCTCCCAACGAGCGATCGGTTTGTTGATACCGTCACTGCAGAAAGTCCGTTGACGGAGCCGTCTCACCTTTGCTTGCAATGCTTCATCACTATCAACGTGAAGTCCTCGAAGGTTTTCTCAGAGTGTTATGTAGGCCTAGTCCGGATGGGGAATGATCCCCGACAGTGGACCCAAGGCTTCTGCTTGCTGCAGATTTCGTAGCGCTCGTGAGTAGTCTGACATTGTCATGTTGAAGGAGAGGATGCTTCATGTGCCGACAAACTCTTTGAATTCGTACTTTTAGTTTTCTGGGAGCCAGAAACTCGTTTACAGCACGCCGTTTCTCACATATCGATATACTTACGTTCAGTGGCGAGGACTGGATTTTGATCTGACGTGCCTTTATTCCTAGAACCGTTTTAGACTGGTACCACGTCTCTCGCCTCAGGTGTATCGCATAATAAGGCATAATATCAGACATTTGTACAAAATGGCGAAGTACTTTTGACAAGCTAGACTGTACCCTTAGGTTTCTGCCTGACCACATCCTTTAAAATCAAGACATATTGGTAAAAAAAAAAAATAGTAAAGTCCTTTTGTGAATCAGAATAGACATCTTAAGTGTAGCTTATCAAAAGTACTTTGCCGTTTTCTACGAATTTAAGATATTAGGAAGGATGGAGGAAGGAAGCTTAGGGTTTAACGTCCCATCGCCAGCTTGGTCATTAGAGACGGAGCTAACGCTCGGAGTTTTAGGTAAGGATGGGGAAGGAAATCGGGCGTGCCCTTTGAAAGGAACCATCCCAGCATTTGCCTGGAGTGATTTAGGAAAAACACGGAAAAGCTAAATCTGGATGACCGGAGGTGGATTTGAATCCCCATCCTCCCGTATGTGATATTAGGCCGCACTACACGATACATATGAGGCGAGAGGCGCAGTACCACTCGAAAACGTTTTTACAAATAAAGGCTCGTCTTGGTCATGGGGGCTGAGTATCTACATGCCCCAAATTACACTTGAAAAAAGTGCAGTTTCGAATTACGCATCACCATATATTAATAAAACTTCATTATTTCTCTCTCTTTCAGTTCTCTTCTTTATATATGTATATATAAGAAAAAAAATGTGCAGGTTAACAAGAATCAACAGCACGCGGCGAGGGGGTGGGGAGGGGGCAGACTGCGGTCGTTATGCATTGCCATGTTATTACATGCTACAATTTGGAGCGGCAGAAGTACTCCATTTGCGCCAGAGAAGCGAAAAAGTAGGCGAGTAATATACATGACGTCAAATACCTCAACCGATAAAAAGTCATAATTTTTCAGCCCGCCCTTTAAGGTCAAGGTTGGCATACGTCCTGGATTTCAAAAGACAGTCCTGTAGTTTAAGAAAGTGCCATGTGTCCCGCTAATACCTGTTCTGGGACGCGATTTATCTCGTATTTTCTCGATTTATCAGTATCGGAAAAAGAATTATTAAGTTACAAGATCACCCGCTAAGGTAATATGATGTTCCATCAAAATTGCCCCAGTCGTCTTAGGGACTATAGGGAAAATACATGCATACATGGATCTTTGTACATACAGCTGCAAGTCACCTTTTTTTTAAATTATTTTTTGACAGTTTTGTTTGTAAAACATAACATACAATCACTTATAGTGCTTTTCATAAAAGTAATAACGTTGTCAGCAAGGCATAGGCTTTATATTTGGATTGGTCATTGATGAAAGAAAGTAGACCCTCCACCAGTTCACTGTATTCAACAAACAGCTACAGCAGTGGCGTAACTAGGACTTGGCTTGGGGGGGGGGGGGGATAGATCTGCTTGACGAGCAAAATTTTAGTCAGCTCCCAGACAGACTACCATCTAACACTCCTGTTGAAGAAACTAATTCACTCCCATCCAATGAAAGCTTATGGTAAAACAGCCCAAGTTTCCAATCTTTACCTTTACCATATTGCTGTTATACGATTTCTTTTTCTATCTCTCTCCCTCTCTCTCTCTTCTTCTTCTTCTTTTCGGGTTCTGGGGAGGGGGGGGGTTCTATCCCCCTCATCCCCCCATAGTTACGCCACTGTGCTACGGAGTACAAGGGCTTAGGCCTACGTAGGATAACATATGGGAATTTGGGTTGGACGGGGTACGGATCGATTAGTCCATCCATCTAATGACTATAAAACACAATGCTAATATTTACAGTGTTCAGTTACGGTACATTTGAGTTTTCTTACGCCTTGGGAATGGCTGTGATTTGCCTGC**AGAAACATTCAGTGAACCTGATCCACATCGAGTCACGTTCGTCGCAGCGGTTCCCTAGCCAGTATGAGTTCATGGTGGAGTGTGCTCCAGGTGGAGACATCGGTGGTGCTGTGACCAACTTACGGCAGAACTCCGCCTACTTCCAGATCATCTCGCGCAACCACAAGGACAACAGAG**GTTAGTTGCCCACAGTAATGTAACGACAGAATCAGATATTAAATTATACCTTACGATTCAATCCTTTAATCTACCACCCTATACCTAATCTTTATGTACCTACCCAATACCTATTGTTCTGTCCTCCATTCTTTCCGTTTTTTTCACTGTCAATCTTTTTATTGATGGCATCGAACTAAACTCCTATCCTTGCAAACCGACTATTTATTTCACTGAAATGACCATTTATTTCACTTTTTATTTCATCGAACTGACTGTCTATCACTTCAAGCCGCTTGTCCATAGTGGCGAGTTGCTGTAATACTACCATAATCAGATCTACTTTACCTTCAGACACTTCATCGTCAGTTGAATAGTTTTCTTTACTCTCTTCAAAATTCCCAGTTTTCATATCTACTGGTAGTTTATTCGTAATGTCATCGAAATTTGCCATAATTGCTGTTTCATCGTTAACCTCAACACTTGTATCGAAAACTTTGTTACACGCCTGTCACGACGCAGTTTGTTGCTAAAGAAAAATATAATATAGGTTACTGATCTTTTTTTCTTTGCTGTCTGTAGATTGTGGTCTTTTTCACATCCAGTATATTGTCCCAGTCTATTTACATGCACAGTCAATTGATCATTGTTTGTTCTTTCTCTGATTCGTAATACCACAGATAACACAGTTATTTATAAATACCTTGCTTTTCAACTGAAAAAAAAATAATGCATTGTTCCAAACCCCCAGCTCAATTCAACCAGTTGAAGTGTCCCAATATTTTGGTTGATTATTGGGAACACTACTATATAGTTCCATTGAAAATTAATTTCTTATAAATCATTCTTAATTTATTTTTTTAAGTATCTTAGTGGAGTCAGTAAATGAATGAAACAATATTTGTCTCAACTTCTTTTATTCAGATGTAGCTCCATCTAACTGACTAATATCAAGTAAAGTCAACATCAACTGAACCAACTTGAACTGAAAAGTAACTGCACATCAGTATCTTAGTTCTATTTACACAATCTTGACAATATGATGTGTTAATACATTGTTGTAATTGTTCAATTACAATGAAAGTTGATGGTTACAGTCCAGTTATTCGTTGATACACGTCTTTTGTATAATATGAACATTATTTCATCCTGAGTTACAAATGTTCATGTGACATACTCTAATTGTGACATTAATAATCATCTGGATATACTTTCAGAAATGTTCATCATCAGCACAAAAATATACAGAGATGATTTTAAAGTTACATTTGTGATCTTTTCTACATACATTTTGGGTTTGAATTAATTTTTTATGATTTGCTTAATTGACAACTGGGACATTGACAACTGGGACATTGACAACTGTCTAGGGTTGACAAACCAAAACAAGACAGCAGAATTATATGATGGTTGTGAATATTGAGACAAAAGCAAACTGTATGTACAGTTTAGTCACTTGATGTACTTCCAAATGTCTACTTTATTTCATCATTGCAAAAATTGGCCCAATTATGTTTTCAATAAATGAAATGAGGCACTCGACCACCTGAAAAAATCTCAGAAGCAAATGACCTCTATATGATGCATTGCAAAGCTTAAAAGCATCCCTATATTAAAGCTGGTCCATTAGATTCTTATCAATTGCCCTAGTTAATATGAAGAGAAATGGCAAGATTGTAATTTTTCCCCTGATTCTTTATGCAAGTTGACCAGTTTCAACACTTAGGGGTGTCATCCTCTGGTCTTTGCTACAGTTGACATTTAACGGATTATTAAATCACTCCAAATATTATGCAGTTAAATGCCAATTGTAGCAGAGACAAGAAGATGACACCCTTAAGTGTCAAAACTGGTCATCTTGCATAAAGAATAAAAATTATGATCTTGGCATTTCTCTTTATATTAATATAATAAGATGGCTTCCTGTGATGAGTAAATAAAGTTAAACTTTCCTAGTGTTTAAATATAATGTTGTTAGCCTATACAAACTATAAGAAACAAGAGTTCAGAATGGATGACACAACTAGAAACACTTTGTCAGAATTAATAGTGGACTGTCACCTTATTCCTTTACTTCCACACATGCCCATTCAGAATCACCCAGCAGAAGTATAGCAGCGATTTATGGAATATTTCATATGATTTC**AGATACTGTTCCTTGGTTTCCACGCCGTATCCGTGACTTGGACAAATTTGCCAACCAGATCTTGTCTTATGGAGCTGAGTTGGATGCAGATCATCCTGGTTTCACTGACCCAGTGTACAGAGCAAGAAGAAAATATTTTGCTGATATTGCGTACAACTACAAACA**GTAAGTCACTTCAATCAATGAAGTATAGTCCTTAACCTACAACATTCATGCAACTGACATTATGAAGAAGGGAAAAAAATAGATGTTAAATATCTTTTTCTCTTCTAATACACGGAAACAAATCAGTATATATTGTACTTCAGGGATACTCAAGTTCTGTTGTAAAAACTGCCTCCAAACTGCAGGACTGTGACCTCAACGAAAGACTTAAAGAGCAATAGTCAGTTGCCAATGCCAAACAAATCTACCCTGTTAGAGCTAAAATGCTTTGTGAATCTCAAATTGGCTCATTTATTCTGTTGAAGCTATTGAAGCACATCTTCTGTGCTCTGAATATCTGCAAAACTGCAGCTTTATTGACAACTGAGAAAATTCCATTTTATTTCTAATCATACCTATACATATTGTTTGTAATTCATACTAATACATAGTTTTATTTCAGTCATGTTGACATTTTTCACACTTGTTCACTATGGGGGTCAGAACCACCTATGATACATAGTTCAGGAGACATGAAATTATAAGCAACGAAATGTCTGAAAATTGCTAGAACATATGATGTGTCAATAACAGTGATACTTTATTCATCTGCTATTTGACAATGACAGCACTTAGTGACTTGCATATAATTTCAAATCTGAAATAAAAATTTTCTCCCTGATGACCTCAACAAAATGATGAAAGGAAAGAAGTTTATTGCTTATTACATTTTCACATTTCATCAAGTACAAGTACAGAATTTGACATGAATTTATTGCCTCTATACTACTAACTGTGTTCATGATACATATTACAGACAGTATGCACATATACCACTCAGGGTACATGCAGAATTATATCATATAGTATGATACATAGCTTAGGAAGTGTGATGTTATTCACTGTACCTTTAAGGTTCATGTAACTATCCAACCATGTATAATGTTTTAATTTATTAAATTGTTACTACTTTTGTGGGTAGTATGAAGCTTCAAAATTAAATGATAGTATTACACATAGTTGAGGAGAAATGACATAATAAACAATGAGATGCATGAAAAATTGCCACATCATATATCAGGTTTAATTTATAAATTTTTTACTACCAACTCTATTTGCAACGTATTTTGCAATTTCCTAAGATGTAACTCTGTATGCCAGAGAAATTACATAATTTTAGGCCCCATTGTTTGGGAGAGTTAACATTATAAGGCTTGAGATGCATGAAAATGAAACTGCAGTTCAGAATTTGCTACAGATACAGGTGAAACCGAGAAAGTTATGGACAGATGTACCTAGGCAATATTTTAAAGCTAGCCCTTGATACATGTAATAAGTTCATTTTAGTCTACAATTTCAGTAACTGTGTAGCATCACTGCTTTCATACACTCCATAAGTTTGAGTCCATTAATATATGTATTTGTGTTCATTTGACTCTTCAAATGCCATTTGACTGACAATATGAGGTAAAGGTTAAAGCAGCTCCTCACACTTGAAATATTACATCAGTGCCATGCAAAGTGGATTGCTTAATACATCACTGACAACATTAACATCAAATTTTCCTTTTATTACCAGTGCTCATTAGGTACTATAGCAATAGATCCACATCGCAAAAATAACTGACATGTAAGAAACCTTTTTTCATAATCCAAATGATTCAAAAAAATATTGACACTCTTGGGATGGTAGCCAATTTCTAGTTGTAATGTATGCACCAAAAGTTTATTTACATTCTTGTCAGCAAAGAACTTCAAAATTTTGTCAACCATAACAAACTAACTACTGTAATAAACTTTTATCACTCACCTACTAAGGATCAACTTATTATCCATTTTAAGCTGTTTATGTATTTTAATATGGCTACTTGTTTAACATAAAGTTGTATAAGTAATGACAAATGGCTGCTTGCAGAATAAGGGCTAAGGCAGAATCCATTTTTCCCATACCTCTAGCAGTGATTTGTGATATTCAAAATACCAAGAATCACCATGACACCAATTTCACATTTACCATGTTTCTTCATGACTGAACAGAGGAATGAAAATGGCTTGTGAGGGGCGATCAAATGTCATATGATATTTTCCTGTTATTTAAAATCACATGTATTTTTGCATGAAACTTCCTTATAGCTGTTACTGAAGTGTTATTTGTATGCTCTTCCTTCCAG**TGGTGAACCTCTTCCTCATGTGGACTACACCAAGGAAGAGACTGAGACATGGGGAAAGGTGTTCCGCGAGTTGACCAAGCTCTACCCAACGCATGCATGTCGTGAGCACAACCATGTCTTCCCACTTCTCATAGAAAACTGTGGTTATCGTGAGGATAACATTCCACAACTTCAAGATGTCTCAAACTTCCTTAAAGGTAAGCAGCACATTTTGTACATTATTTTTAAAGCTCTGAATAACTTCATCAAAATCTACTGAAGGTTTCACACTCAATACCTAAGTTCCAAATACATATGGAAAGATGTCACTAAACATCCACTCTGGCCCATTCATTCAGACTCATGTCTAAATTTATTCACATTACCTAAAATTACTTATCTTCAGTTAATTTATTGGTGATGTATTTTCAAGTGGCTAGTCCACATATCTGTGTATCAAGGTTGTAAATTATTAGTTACAGTACCAGGAACTACCAGATTATCAGTAGTTCATTGAATAGGAGTCCCACAAGAATTGTTGTCACCATATGCAAGCCTAATCCTGTGATAATGTTAGCATAGTGGGACTATAATGTTCATCTCAATATGATGTGTTGCGTTTGTTGGCAGACTGCACGGGTTTCACACTGAGACCAGTAGCTGGTCTGCTGTCATCACGGGACTTCCTGGCAGGGTTGGCTTTCCGTGTCTTCCATTCAACACAATATATCCGACATGCCAGCTGCCCACTATACACCCCAGAACCTGATGTCTGCCATGAGCTGCTAGGCCATGCACCACTCTTTGCTGACCCTGCATTTGCACAGTTCTCACAGGAGATTGGTCTAGCCTCCCTTGGTGCACCAGATGATTACATCGAGAAGCTTGCAACAGTGAGTATTGTAGCTATCTTATAGGCTCCAAGTAGTATTGCACATTTGCTGACTAGGCTTTTAATGAAAAAGTCACCCACTAGAAGTAAAGTTACTATAGTCTCTATATTCTATAAGTCCTTCAGTATTGTCATTATCAAACAACTATACAAAAAGATTTAAAAAAAATACATAGCCATGTATACAATATTTAAAAAGAGTACATTTGTGCTCACTGTAAAGACAACACTCTGAGCAGCATAGAGGCACAATAAGATCATCCTAACGAATGTTTGGCCAGTCTTAATGAGAAAAACAAGATAAACAAACACATAAATTCATACAGTCATATACATACAACTCATGTGCATGCAAATGCATTGCATGCACGCCTTTTCTAATGAAGGCCTGGCTGAAAGCTCATTTGAACTTTTTGCCTGCCTGCTTCTTGTCATGTCATCTTAACGGTGAGTAACAATACATAATTTCCACAGTATTGTAGATGTACCAACCTGGACTTATATAATTGTTTGATTTAACCATAAGTAATGCAATGAAATTAGGTTACAAAGGACTACCTGTCATTTGCAACATCCAGGTTTCTAATAGATAAACATAAATGGGAGTCAAATGAAAACACAGTGACTGCTTTGCACCTAGACATACATCTCAATATTCTTGAGGAAATTGTAAACATTATACTGACAATTCATACAATACTCTAATTGACAGATTGGTTCAACCATAAGTGACATACTGGATATGTGGTTTCACACCTCTCTCATGGGACACTTGACAACTACATGAAAAAGCAAACTTTATAAGTCACATAATGTACACTGCCAAAACTTCTGCGATTTTTTCACAAATATATTTTATATAAACTAATTGTTTTGAAATGCAATATTGTAAGACAAGGCCAGTAATATATAAGAGAGAGAGAAAAGATCGCAAGACAACCTGTCCAAAAAACTAAGGTTATACTAGTATCTATTTCACATCATTTCTAATTTACCTTTTGTTAGAACCTATCTACTCTATACATTGTTATTTTTCCACAATATTTTTTATTTTCATAAAAAATTCAGACACTACCAATACTATTTTTACATTTTATGTCATATAATAGAGATTATCATAATATTTCTGTGCATAGCAATTTTAACCCCAAAGGTCACGTTGTGTGGTGTAATGTGAGATGAAGATACACTATTATCCACCTTGATTGTGTGATCAACAAATCTGTATATTCTCATAAAAAATAACAATGAACTCCTTATATCTGTATTATCACTCTGTATTAAATATTTAACTTTCTGAAGTAGGGAGCATATGACCTTTTTGTTAGTGAAATATACTGCTGCAAGTGATAACTTACAAACAGCTAAAGAACTACAGTTCAGATTTTATATCATACATGCTAATGAATGCATATATGTATCTATACTGTTTCTATCAATGGAAGGTTTATATTGGAATTCAATAATATTATGAGAATAGGGTGCAAATTGCCCCACAGATGTCAATGAACCACAGAAAAGCATGTAGAAAAGACAGTCACATTTGTACAATTTAACTTTTAGACCCAGATTTCATAAGAAATGAATGCTTACACATTCATAAAATCACAGTGGCACTACTCACATACATGACTACCATCTCCAGTATATATATTGTGAGTCAGGATAGTGGCCAGAGAAGGCAGACATGTATGGATGAGTTGTTCCTGTATGATTGGTGGAGGTTTATAGGATCGGGTCGGATAAGACACGAACACTCCAACTTTCATACACTGACTTTATTATAACACGTTTACACTATACATGTCAGGCGGGAACTGGTGTTTCCGCTTCCTTACAGCAAAGACCTTTCGTAACACAGAAAGATACTACACTCGTCCAAAGTTGTTAGGTATCGATTCCCACATAGCCCCCCCCGGCAGTATGGAGTACATC**TATTCATGTGACGTATGTTCTTTCCTGATGAAGGCTATGCCCAAAAAATAAGTTTTGTGAATGTGACTGTCTTTTCTATATGCCTATTTGTAGCTTAGAAATGGTCTCTGTGGTGAGCTGCAACCTTTCCCCATTATACTTTCTGTATAGTATGATATCACCATCTTGTGTTGCAATAAGTGTTACAGTACATCTACACTGCAAGCCACCCACCATGTGATGTGTGGTGAGGGTTTATCATACCATTATTCCAACATGGTATATGAGGGGGAAAGGGGGGATGAGACAGTAAAGTACATTTAATACACTACATATAAATGTTAAATGTCTAAATAATATTCCTTCATTTTTTGTTTTAG**TGTTTCTGGTTCACTGTTGAGTTTGGACTATGCCGTCAAGACGGCCACTTAAAAGCTTATGGTGCTGGTTTGCTATCTTCCTTTGGAGAGCTGCAGTACAGCCTGAGTGGAAAACCAGAACTAAGACCATTTGAACCATCAAAGACTGCAGAACAGAAATATCCTATAACAGAGTATCAACCAGTTTACTTTGTTGCAGAAAGTTTTGAAGATGCAAAAGAAAAAATGAT**GTGAGTAACAAATAACAATCATCGTTAATTTTATAATCACTGGCCAGTAATTGCTTTCAGAATGTGGAACTTTTAGTAGGTTTCATAGAAACACATAAAAAGATGTTGTGTTTATGGTGTTCCCAAAAAATTAAATCCTTCATTGATTCATGGGTGTTGCATTGAATTGAGTTGTGAGAACAACCTAGCCTCATTTCTCCACTTTTCTACTTAACCACAAAATACATTAGCAAGTCACAACAGCACCCAAAGATGAATATCAGCTATTTTATTGAAGTGTCAGGCTGTTTTCACAACACAATTCAATGTAGTACCTTTGATGCACTGACTCACATTAAAGTATGTTACATGTTTCTGCTAGAAATAACGAGCTACATTTTAATGTGACTACTAACCACACTCACTTGTCTCTTCACGAACTTAACAAGGGTTTTTTTTCTCTCTCTCTCATTTCATATGTGTAAGTGTGTGTGTGTGTGTGTGTGAGAGAGAGAGAGAGAGAGAGAGAGAGAGATGGAGGGAGGGGAAAGAGAGAGTAATTTAAAATTATGAAACAGAATTGCTGACCTCCACTTATCTTCAAGGCACAAATGTTCAATACTTATGATAAACACTTGATATGGTGTCTGTTCTTTGGGACATGTCTAAAAGAACAGACACCAAATCAGTCATTGCACATATGCACTAAAAGGAAGTTGACAATTTCTGTGAAGATGCACAGATATGCTTGAGTCTTTGTGGGACTCCAGTAGAGGCTGTGAGCAAGAAGAAACTAATGGACAGGGGGCACTATGATCATAGTGTGCTATAAGTTGAGAATTTGGGCAGAAAGGGAAGCACACCTGGATAGCCTAATAGGTTAAGATGAATACTGGAGTGAAGTGGAAAATTCAGGTTCAAATGGAAGTCCAGCACCAATTTTCAGTTTTGCTGTTGAATTTATTTAAGTGCGCTTAAACAGTTGAAATGTTTCAATATTCCAAATTAACTAATCAGAGTACATCTATAGAAGATAAATAACTGCCAGTCAGCAATTCTGAGTAATACATTTATACTGTTCCAGAGAAAATATGATTTAATGTTTTCTTGTAGTCTGTGTGTAACCATTCAATTTTTTTTTCCACAG**CAAATATGCTCACACAATTCCACGACCTTTTGGAGTGCGCTACAATCCATACACACAAAGTATTGAAATACTGGATTCAAAGCCTCAGATACAAACCTTGGTAGACAATATTAATGAAGAAATGCAAATTTTAATGGATGCCTTGCGAAAATTGTAAATCAGCAAACATAGTTCCTGATTTGTACCATACCAAAGCTGTAATATGTAGATAGATGTGTTAATATAATATTTCAAAGATCATAGATCAAACAATAA**

# Supplementary Figure S1. Full-length sequence of the *PAH* locus. The green boldface represents the exon sequence of *PAH*. The blue boldface with underscored characters represents the sixth exon of the *PAH* gene that is covered in the *PAHAL* and *PAHAL¯* sequences. The bold text with underscored characters represents the same sequence of *PAHAL* and *PAHAL¯*. The boldface with yellow shading indicates the sequence of the embedded *Gypsy* element in *PAHAL*. The red words indicate the specific sequence of *PAHAL¯*.

**
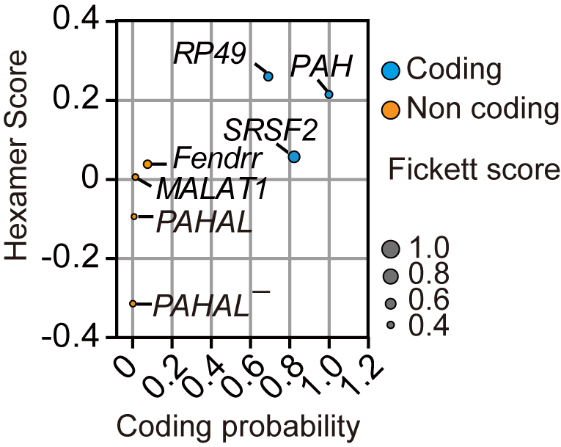
**

# Supplementary Figure S2. Coding potential of the *PAHAL¯* transcript and reference transcripts through the analysis of CPAT algorithm. In the references, *MALAT1*, *Fendrr* and *PAHAL* are lncRNAs, and the others are protein-coding genes.
