# Supplementary figures and images for: A Gypsy element contributes to the nuclear retention and transcriptional regulation of the resident lncRNA in locusts
Source: RNA Biol. 2022 Jan 22;19(1):206–20. doi: 10.1080/15476286.2021.2024032 (PMC8786324; doi:10.1080/15476286.2021.2024032)

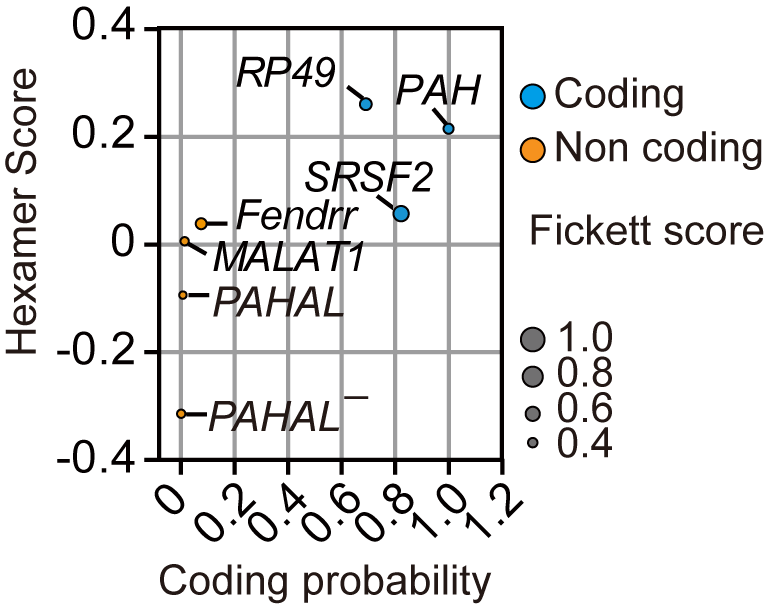

Supplement: Supplemental Material [file KRNB_A_2024032_SM3081.zip › supplementary/Supplementary Figure S2.tif]
